# Understanding the Impact of Symmetrical Substitution on the Photodynamics of Sinapate Esters Using Gas-Phase Ultrafast Spectroscopy

**DOI:** 10.1021/acs.jpclett.3c02134

**Published:** 2023-09-22

**Authors:** Jack Dalton, Josene M. Toldo, Florent Allais, Mario Barbatti, Vasilios G. Stavros

**Affiliations:** † Department of Chemistry, University of Warwick, Gibbet Hill Road, Coventry CV4 7AL, U.K.; ‡ Aix Marseille Université, CNRS, ICR, Marseille, France; § URD Agro-Biotechnologies Industrielles, CEBB, AgroParisTech, 51110 Pomacle, France; ∥ Institut Universitaire de France, 75231 Paris, France; ⊥ School of Chemistry, University of Birmingham, Birmingham B15 2TT, U.K.

## Abstract

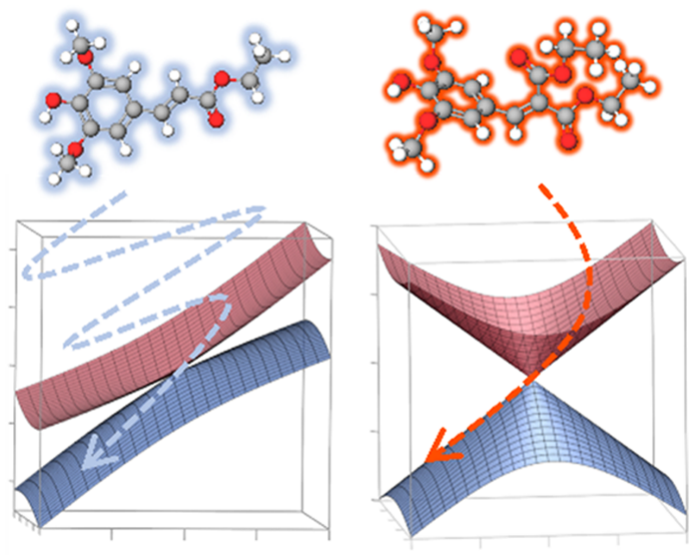

Two model biomimetic systems, ethyl sinapate (ES) and its symmetrical analogue, diethyl 2-(4-hydroxy-3,5-dimethoxybenzylidene)malonate (or diethyl sinapate, DES), are stripped to their core fundamentals through gas-phase spectroscopy to understand the underlying photophysics of photothermal materials. Following photoexcitation to the optically bright S_1_(ππ*) state, DES is found to repopulate the electronic ground state over 3 orders of magnitude quicker than its nonsymmetrical counterpart, ES. Our XMS-CASPT2 calculations shed light on the experimental results, revealing crucial differences in the potential energy surfaces and conical intersection topography between ES and DES. From this work, a peaked conical intersection, seen for DES, shows vital importance for the nonradiative ground-state recovery of photothermal materials. This fundamental comparative study highlights the potential impact that symmetrical substitution can have on the photodynamics of sinapate esters, providing a blueprint for future advancement in photothermal technology.

Photothermal materials absorb light and convert the absorbed energy into heat via nonradiative relaxation pathways. They are paramount for various new applications, including cancer therapy;^1−3^ clean water production;^4−6^ and more recently, crop protection.^7^ For these materials to achieve optimal photothermal conversion efficiencies, the system must relax from an initially excited state solely through a nonradiative decay mechanism without forming long-lived excited states, detrimental photoproducts, or both. Moreover, to enhance the photothermal performance, these systems must have excellent light-harvesting capabilities by strongly absorbing light.

The naturally occurring sinapate family of molecules are ideal candidates for new photothermal materials due to their intense ultraviolet (UV) absorbance and efficient nonradiative relaxation mechanisms while avoiding harmful side reactions and detrimental photoproducts.^8−16^ However, the impact of novel molecular functionalization, such as symmetrization, on the fundamental photophysics and photochemistry of sinapates remains unknown.

Ethyl sinapate (ES, see Figure 1) is one of the simplest sinapate esters that one can use as a starting point for biomimetic photothermal molecules. Following photoexcitation to the optically bright S_1_(ππ*) state in a weakly perturbing solvent, as cyclohexane, ES undergoes *trans*-*cis* isomerization toward an S_1_(ππ*)/S_0_ conical intersection (CI) with subsequent vibrational relaxation to either return to the starting *trans*-isomer or form the *cis*-isomer photoproduct.^17^ The formation of the *cis* photoproduct for ES in solution results in a loss in peak absorption.^17^ Therefore, an intuitive solution is to remove the potential of *cis*-isomer formation through symmetrizing the ester moieties across the acrylic double bond. This approach with ES generates diethyl 2-(4-hydroxy-3,5-dimethoxybenzylidene)malonate (diethyl sinapate), abbreviated DES hereafter (see Figure 1). The photorelaxation mechanism for DES in cyclohexane follows a similar route to ES, with a rotation around the acrylic double bond, albeit with a nearly 10-fold reduction in the corresponding lifetime that could be attributed to an aborted photoisomerization process due to symmetrization.^18,19^

**Figure 1:**
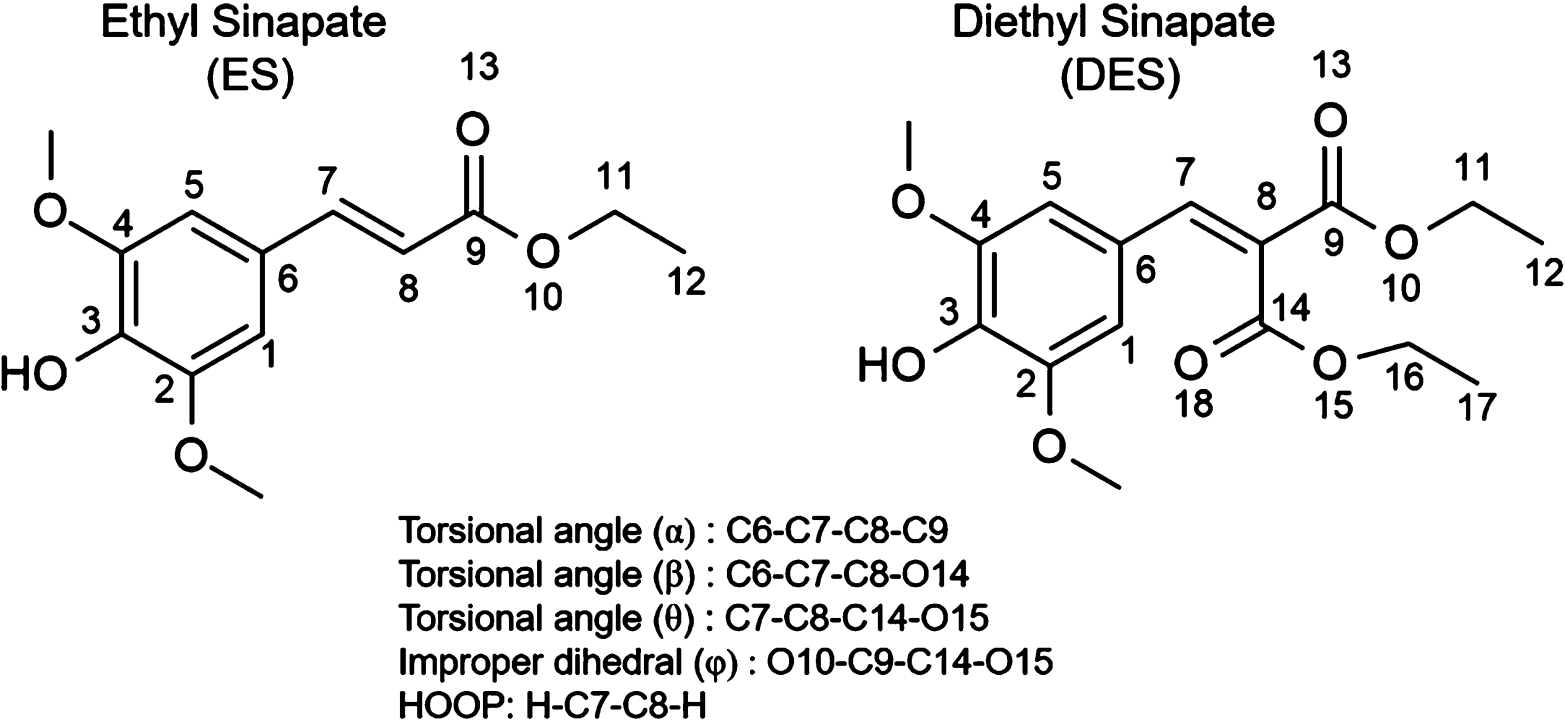
Chemical structure with atom numbering of ethyl sinapate (ES) and its symmetrical analogue, diethyl sinapate (DES). Below are the main torsional angles discussed within.

To further develop our foundational understanding of how the photodynamics suddenly change with varying functionalities and, ultimately, environment complexity, we first need to understand the excited-state dynamics of ES and DES without the influence of external perturbations—in the gas phase. Methyl sinapate (MS), a close ES structural derivative, has been extensively studied in gas and solution phases.^20−27^ Since the ester group is shown to have a negligible effect on the initially populated S_1_(ππ*) state, the excited-state dynamics from this state should be similar.^26^ Baker et al. proposed that following excitation to the S_1_ state, MS undergoes intramolecular vibrational energy redistribution (IVR) within 3 ps before intersystem crossing (ISC) to a nearby triplet state, most likely T_1_, in 30 ps and persisting in the triplet state beyond 1 ns.^27^ Further nanosecond pump–probe investigations on MS confirm the presence of a triplet state that persists for ∼30 ns.^21,22^ However, despite these extensive studies, none have monitored the molecule’s potential energy on the ultrafast time scale, and thus, triplet state formation within 30 ps cannot be confirmed.

In this Letter, we focus on uncovering the ultrafast photodynamics of ES and DES in the gas phase to answer two questions: (1) Does symmetrizing the ester moieties across the acrylic double bond for ES dramatically affect the dynamics in the gas phase, as seen for the solution phase? (2) Is a triplet state involved in the relaxation mechanism of ES in the gas phase? To address these questions, femtosecond (fs) time-resolved ion-yield (TR-IY) and time-resolved photoelectron (TR-PE) spectroscopies are used to probe the subsequent excited-state dynamics that occur after populating the S_1_(ππ*) state of ES and DES in the gas phase. The TR-IY results provide insight into the lifetimes at which the photodynamical processes occur, while the TR-PE results primarily provide insight into the changes in energy that these processes induce. Additionally, we explore the potential energy surfaces (PESs) and CI topographies using computational methods to account for geometrical and topographical changes driving the photodynamics of ES and DES. Based on these results and the literature discussed above, we rationalize the relaxation mechanisms of these molecules in the gas phase and uncover the lifetimes at which these processes occur. These results ultimately aim to demonstrate the potential merits of molecular symmetrization.

*Experimental Gas-Phase ES Results*. The gas-phase photodynamics of ES were investigated using TR-IY and TR-PE spectroscopies. Since no prior spectroscopic ES studies have been conducted in the gas phase, the S_1_ ← S_0_ excitation wavelength was inferred from structurally similar molecules that differ only in their ester group functionality, such as MS and isopropyl sinapate (IS). Frequency-resolved work by Dean et al. found the gas-phase S_1_ ← S_0_ band origin of both MS and IS to be 322 nm.^20^ Thus, this wavelength was chosen for the photoexcitation of ES. Following photoexcitation to the S_1_(ππ*) state, ES’s dynamics were tracked with a 240 or 200 nm ionizing probe. Figure 2a,b shows TR-IY transients with 240 and 200 nm probes, respectively, and Figure 2c shows the TR-PE transient with a 240 nm probe with its corresponding electron kinetic energy (eKE) false color heatmap shown in Figure 2d (selected time delay eKE spectra are shown in Figure S1). Panels c and d of Figure 2 are both extracted from photoelectron images, of which a raw and reconstructed image slice at 200 fs is presented in Figure 2d, inset. The photoelectron images show a near isotropic, time-independent angular distribution of photoelectrons (β_2_ ≈ 0; see Figure S2). A similar finding was found for DES (see Figure 3 and Figure S3). The angular distribution of the photoelectrons is beyond the scope of this work and will not be discussed further.

**Figure 2:**
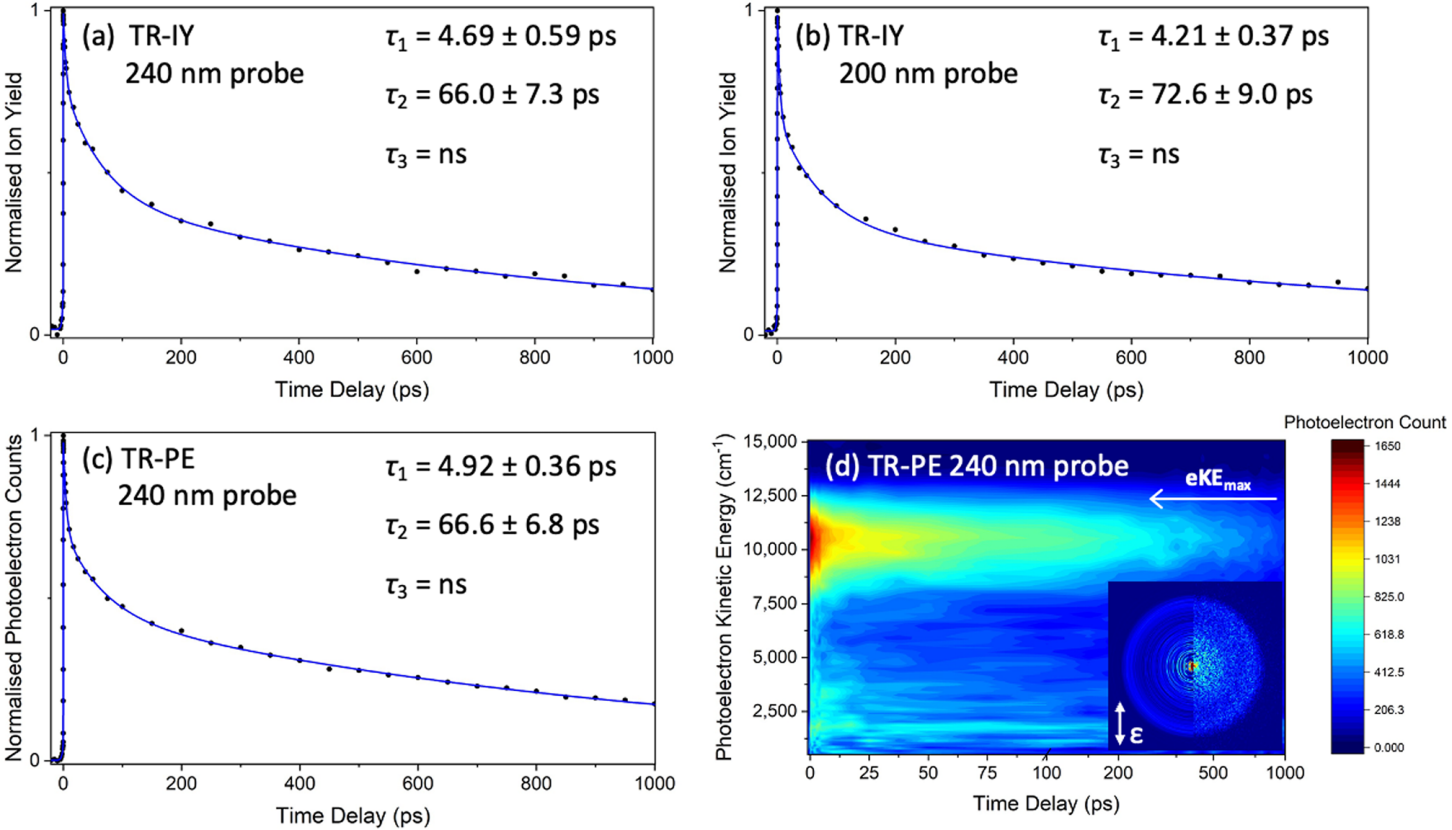
Gas-phase time-resolved ion-yield (TR-IY) and time-resolved photoelectron (TR-PE) transients of ES photoexcited at 322 nm. (a) TR-IY at 240 nm probe. (b) TR-IY at 200 nm probe. (c) TR-PE transient at 240 nm probe. (d) Corresponding electron kinetic energy (eKE) false color heatmap for panel c showing the eKE regions contributing to the signal intensity (the eKE was smoothed with a moving average of 4). (d) Inset: right half presents the recorded image, while the left half presents the reconstructed slice through the original three-dimensional (3D) photoelectron distribution at Δ*t* = 200 fs (the double-headed arrow indicates the electric field polarization of the laser pulses). The white single-headed arrow in panel d indicates the expected eKE_max_ based on the ionization potential of methyl sinapate, discussed in the text. The blue traces in panels a–c are fits with three exponential decays in the positive time delay and one in the negative time delay; forward lifetimes (Δ*t* > 0) are shown for their respective transient. The pump and probe are parallel with respect to one another and in the plane of the detector.

**Figure 3:**
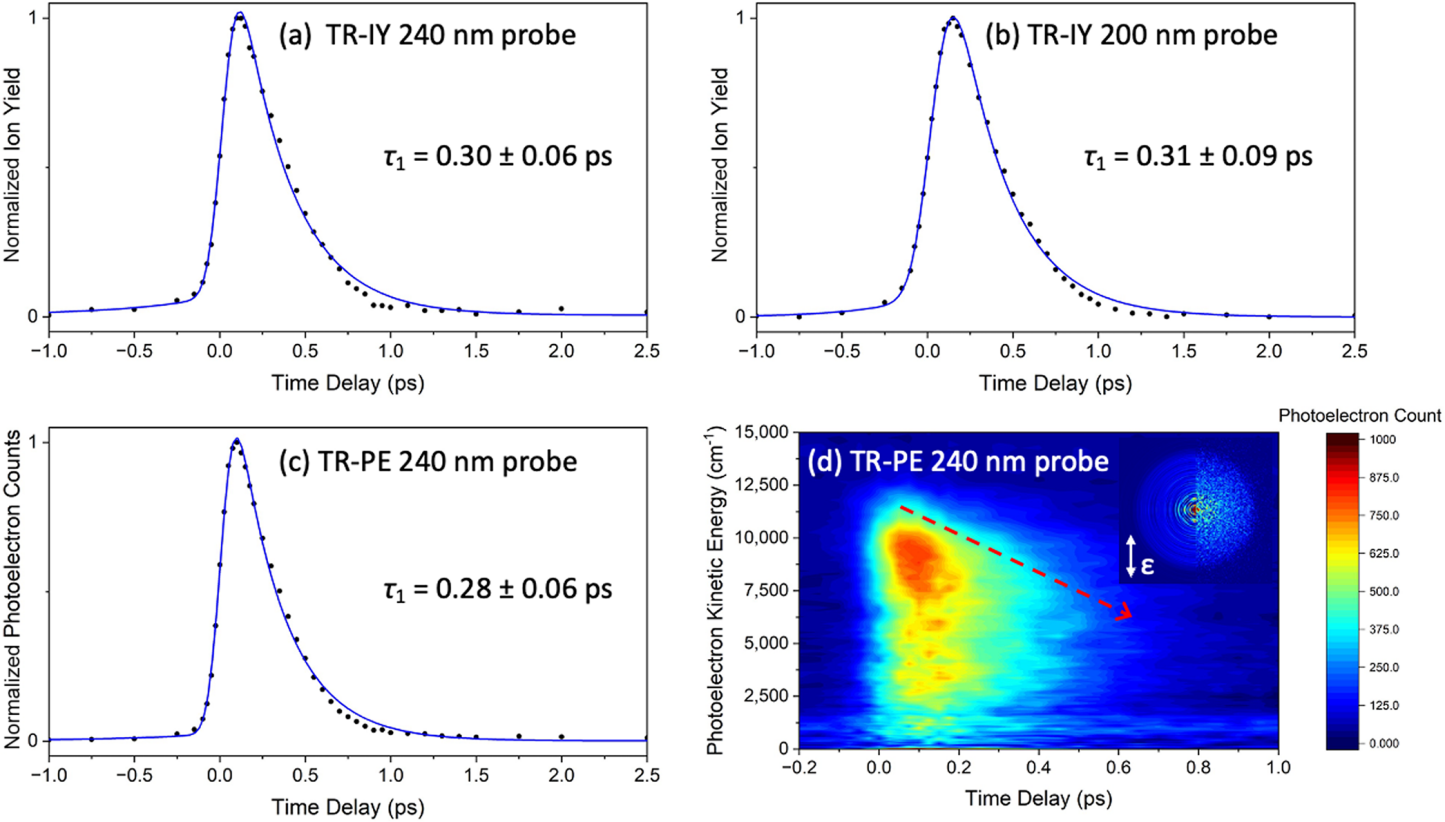
TR-IY and TR-PE transients of DES photoexcited at 325 nm (note the difference in the time delay scale compared to that in Figure 2). (a) TR-IY at 240 nm probe. (b) TR-IY at 200 nm probe. (c) TR-PE transient at 240 nm probe. (d) corresponding eKE false color heatmap for panel c showing the eKE regions contributing to the signal intensity (the eKE was smoothed with a moving average of 4). The red dashed arrow highlights the dramatic energy shift. (d) Inset: right half presents the recorded image, while the left half presents the reconstructed slice through the original 3D photoelectron distribution at Δ*t* = 200 fs (the double-headed arrow indicates the electric field polarization of the laser pulses). The blue traces in panels a–c are fits with one exponential decay in the positive time delay and one in the negative time delay; the forward lifetime is shown for each respective transient. The pump and probe are parallel with respect to one another and in the plane of the detector.

Overall, it is clear from these data that the excited-state dynamics of ES extend beyond the maximum time window of our experiment (1 ns) with no change in the kinetic energy distribution. To gain a quantitative understanding of the photodynamics, the transients were fitted with a parallel (all dynamical processes starting at a pump–probe time delay, Δ*t*, of 0) exponential decay model with three forward (Δ*t* > 0) and one reverse (Δ*t* < 0) exponential decay, in accordance to a similar approach used previously in our group (further fitting details can be found in the Supporting Information).^28^ All three data sets returned time constants of τ_1_ ≈ 4.5 ps, τ_2_ ≈ 70 ps, and τ_3_ > 500 ps.

*Experimental Gas-Phase DES Results*. There are no prior gas-phase studies on DES or structurally similar molecules; therefore, a pump wavelength dependence study for DES was carried out to attain an approximate S_1_(ππ*) origin band excitation wavelength of 325 nm (see Figure S4). Analogous to ES, Figure 3a,b shows TR-IY transients when probing at 240 and 200 nm, respectively, and Figure 3c shows the TR-PE transient at 240 nm probe with its corresponding eKE false color heatmap shown in Figure 3d (the raw and reconstructed image slice at 200 fs is presented in the inset, and selected time delay eKE spectra are shown in Figure S5). A single forward and single reverse decay were fitted to each of the three transients, returning a positive time constant of ∼0.3 ps. We acknowledge that modeling the transients with a single forward decay does not fully capture all transient features. However, modeling the data with more than one forward exponential decay in a parallel or sequential decay model does not yield an improved result (a sequential model captures more transient features but results in exceptionally large errors; Figure S6). As such, we have adopted a more qualitative approach for DES to avoid overinterpretation of the data. Furthermore, the eKE heatmap in Figure 3d displays a steep shift in eKE (highlighted by the red dashed arrow), implying large changes in potential energy as DES relaxes. Nevertheless, all transients for DES fully decay by ∼1 ps.

*Theoretical Gas-Phase ES and DES Results*. The PES of both molecules was explored using XMS-CASPT2, as described in Computational Methods. The main computational findings are presented in the present Letter; further supplementary details are presented in Figures S9–S16 and Tables S1 and S2. Figure 4 shows the remarkable differences following the excitation of ES and DES to optically bright 1^1^ππ* (represented at the top of Figure 4). ES (a) features a minimum on the S_1_ surface that is not observed for DES (b). As previously discussed,^29^ the relaxation from the initially excited ES geometry is driven by changes in single and double bond patterns, yielding a planar minimum slightly more stable than the initial structure (Figure S9). The S_2_ state, with ππ* configuration and ∼10 times smaller oscillator strength, lies energetically close to the S_1_ state, but this energy difference increases under optimization (Table S1). The internal conversion to the ground state is driven by a torsion around the C7–C8 double bond (Figure 1).

**Figure 4:**
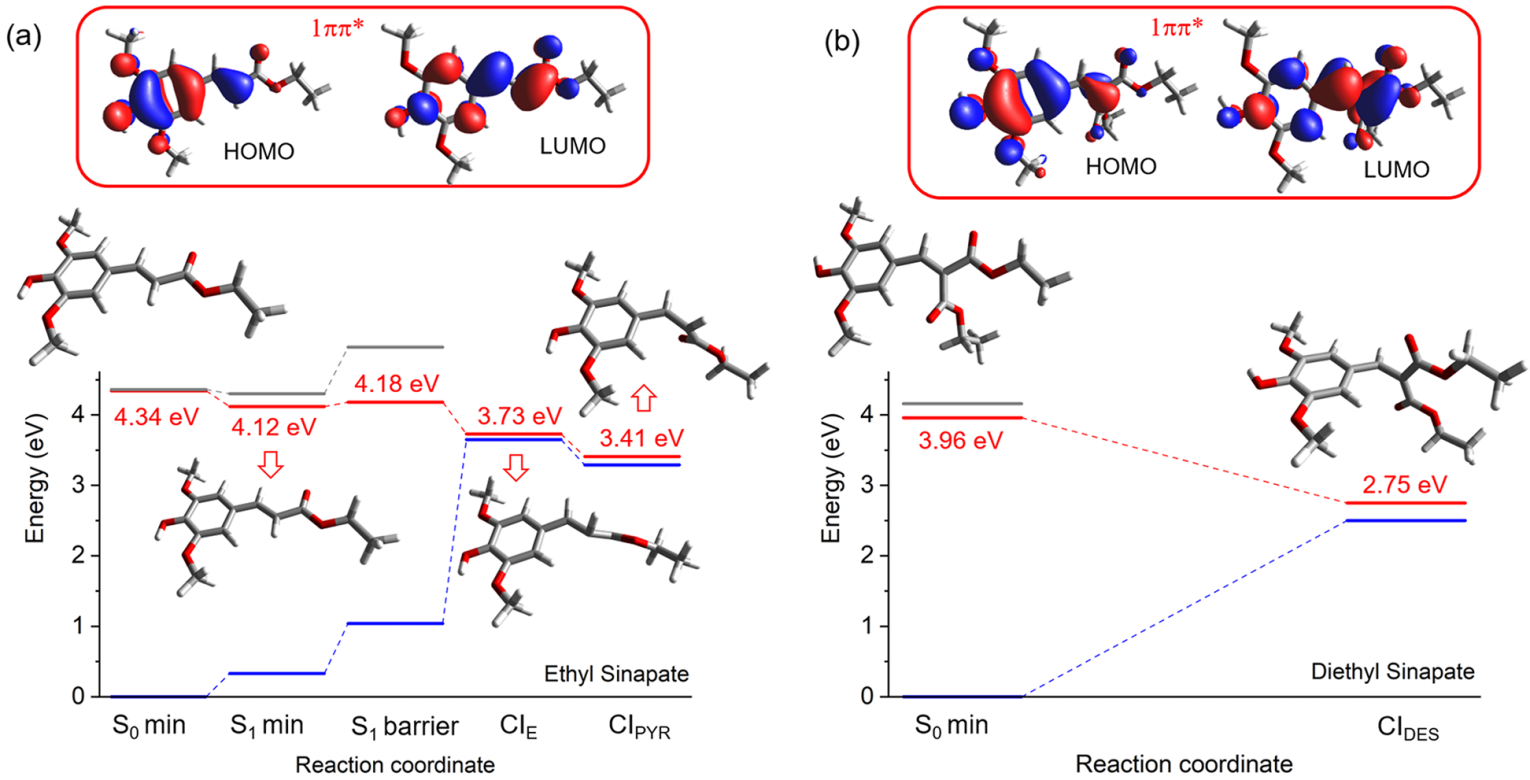
Relative energies and geometries of the S_1_ vertically excited and minimum and conical intersections located for ES (a) and DES (b). The orbitals representing the main configuration of the S_1_ state are given at the top. Blue, red, and gray lines represent the S_0_, S_1_, and S_2_ states, respectively.

The geometrically and energetically closest located S_1_/S_0_ CI to the S_1_ minimum (CI_E_, Figure S9) in ES is only partially twisted (torsional α angle defined in Figure S9, 131°) and presents a pyramidalization on the C8 atom due to a hydrogen out-of-plane (HOOP); the minimum energy CI (CI_PYR_, Figure S9) has a torsional α angle of 104°. These CIs are likely connected through an intersection seam, and due to geometrical proximity, one may expect that most of the population is mainly deactivated via CI_E_. We discussed the other possible CIs in a previous publication.^29^ No significant barrier was found connecting the S_1_ minimum to either of the CIs (see also Figure S16). In turn, DES shows no minima on the S_1_ torsional reaction coordinate leading to photoisomerization. Following excitation, the optimization of this state leads to a ∼90° twist of the C7–C8 double bond (see Figure 1 and Figure S10), characterizing a CI with a significant energy stabilization (1.2 eV) compared to its vertical excitation. Thus, the symmetrical substitution facilitates the torsion and drives the molecule toward the CI via a steep downhill pathway. Furthermore, one can also notice that the additional −OCOEt group is out-of-plane in the S_0_ geometry but coplanar with the other −OCOEt group in the CI geometry (Figure S10).

Hence, the potential energy curve governing the isomerization of ES is considerably flatter than the corresponding curve for DES and involves less pronounced structural modifications (see Figure 5). Nevertheless, there is no significant barrier to the isomerization of ES; therefore, the results presented so far may be insufficient to account for the considerable differences in the population decay experimentally observed. To further investigate this difference, we have also characterized the topography around the S_1_/S_0_ CI, which plays a crucial role in photochemical selectivity and dynamics.^30−32^

**Figure 5:**
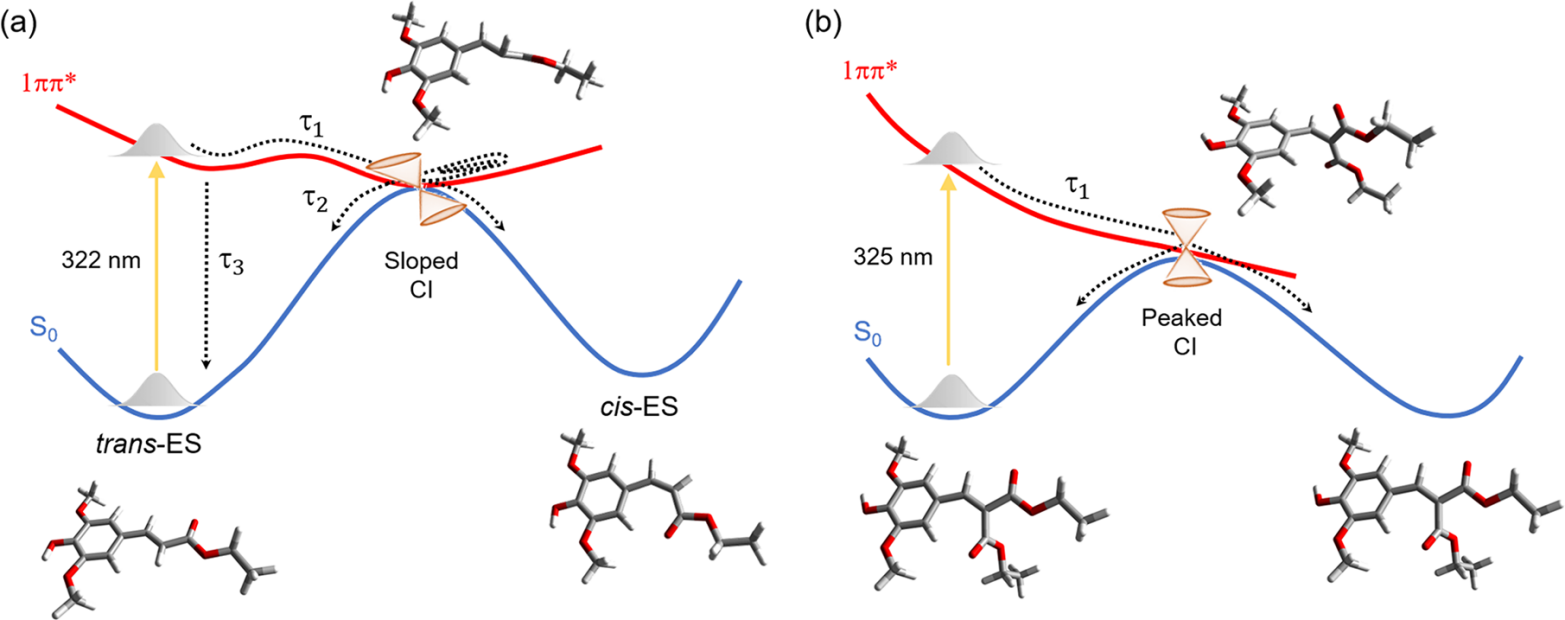
Schematic for the potential energy surface of (a) ES and (b) DES. Yellow arrows represent S_1_ ← S_0_ photoexcitation. Black dashed arrows represent the relaxation processes proposed for the lifetimes found for ES and DES in Figures 2 and 3, respectively. The S_1_/S_0_ conical intersections (CI) are depicted by orange cones. Molecular structures are shown for ES and DES in their ground states and at their conical intersections.

The resulting CI topographies and the two most effective coordinates promoting the internal conversion,^33^ the difference gradient vector (*g⃗*) and nonadiabatic coupling vector (*h⃗*), are shown in Figure S15. The CIs were classified as sloped or peaked according to the parameters introduced by Yarkony^33,34^ and presented in Fdez. Galván et al.^35^ (Table S2). The local topographies of the two CIs are quite different: they are sloped for ES but peaked for DES, indicating significant variations in the photodynamics. A peaked CI is more efficient than a sloped CI in directing the excited-state population toward the intersection point and moving away from it onto the ground-state surface.^30,31^ On the other hand, in a sloped CI, the population is not strongly directed away from the intersection, and as a result, the probability of S_1_ ← S_0_ up-funnelling (i.e., recross to the excited state) is much larger.

*Discussion*. We begin by discussing the photodynamics of ES. For this, it is useful to consider its closest structural derivative, MS, studied by our group.^27^ The TR-IY transients for both ES (Figure 2a,b) and MS are remarkably similar, yielding three commensurate lifetimes.^27^ From the TR-IY transients alone, one would infer the same relaxation mechanism proposed for MS, that is, IVR along the initially populated S_1_(ππ*) state (τ_1_), before ISC to a nearby triplet state (τ_2_), and remaining in that triplet state beyond the experimental time window (τ_3_).^27^

However, incorporating the photoelectron kinetic energy distribution data in Figure 2d brings into question the aforementioned mechanism. It is clearly evident that the kinetic energy distribution in Figure 2d remains constant as a function of time delay, suggesting that excited-state dynamics seen in the transients reflect the decay from the initially populated S_1_(ππ*) state, without populating a triplet state, as previously thought.^27^ As such, we propose an alternative assignment to that of MS for the lifetimes of ES in the gas phase: upon excitation to the S_1_(ππ*) state, the molecule undergoes IVR in the excited state within ∼4.5 ps (τ_1_) and subsequent internal conversion through an S_1_/S_0_ CI within ∼70 ps (τ_2_) (see Figure 5 for schematic). As this mechanism mimics that seen for ES in the solution phase, the path taken along the S_1_(ππ*) PES toward the S_1_/S_0_ CI is likely to involve *trans–cis* isomerization around the acrylic double bond for ES in the gas phase.^17^ This kinetic energy distribution also implies that the topography of the S_1_(ππ*) state PES is very flat, which is supported by our computational calculations.

The remaining lifetime, τ_3_, is assigned to a trapped population in the S_1_(ππ*) state that decays beyond the 1 ns experimental time window of our experiment. This assignment explains the lack of change in the kinetic energy distribution as the molecule decays (Figure 2d) and is qualitatively consistent with the S_1_(ππ*) excited-state minimum detected computationally. Emission quantum yield measurements of ES were carried out in cyclohexane to observe emissions resulting from the trapped population in an environment as close to the gas-phase environment as possible. However, a negligible emission quantum yield was found (data not included). It is worth noting that after our 1 ns experimental time window, trapped population may undergo ISC to a nearby triplet state, representing a minor nonradiative decay pathway as seen in previous nanosecond studies.^21,22^

We close our discussion of ES with a survey of its maximum eKE (eKE_max_). The eKE_max_ in Figure 2d can be calculated from the following equation:
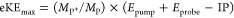
1where *M*_P^+^_ is the mass of the parent ion, which is approximately equal to the mass of the neutral parent molecule (*M*_P_), *E*_pump_ is the energy of the pump laser pulse (322 nm/31,056 cm^–1^), *E*_probe_ is the energy of the probe laser pulse (240 nm/41,667 cm^–1^), and IP is the ionization potential (IP). Since the IP of ES has not been reported in the literature, the IP for MS was used as an estimate (60,291 cm^–1^).^22^ For comparison, the calculated IP for ES is 60,523 cm^–1^ at the DFT level and 57,588 cm^–1^ at the XMS-CASPT2 level. The eKE_max_ obtained using eq 1, 12,432 cm^–1^ (indicated by a single-headed white arrow in Figure 2d), coincides well with that observed in Figure 2d, albeit slightly underestimated. This implies that the ester group functionality of sinapates has little effect on IP.

We now move to discuss the gas-phase photodynamics of DES. From Figure 3, it is clear that relaxation from the S_1_(ππ*) state of DES is ultrafast; the excited-state population has fully decayed within ∼1 ps, with an apparent total relaxation lifetime of DES in the gas phase of ∼0.3 ps. This is over 3 orders of magnitude quicker than its nonsymmetrical partner, ES, echoing the dramatic lifetime reductions also seen in solution.^17,18^ The sheer speed of this relaxation suggests that the molecule’s dynamics occur largely along an unstable S_1_(ππ*) PES toward an easily accessible CI, driving the population to the electronic ground state (see Figure 5 for schematic). Additionally, the identical dynamics observed between the 240 and the 200 nm probe (see Figure 3) rule out the possibility of ISC to a nearby triplet state, as the 200 nm probe can observe dynamics on states as low as ∼10,000 cm^–1^, i.e., within the triplet manifold (see below for the IP). The calculations confirm a steep downhill pathway from the initially excited S_1_ state toward the CI with the ground state (Figure 4), which, combined with a favorable peaked CI (Figure S15), can explain the dramatically shorter lifetime of DES. As proposed for ES and DES in solution phase and analogously to ES in the gas phase, the path taken along the S_1_(ππ*) PES involves a twisting in the acrylic double bond. However, as previously speculated for DES in solution, it is unclear if the torsional motion is complete or aborted.^18^

The eKE false color heatmap for DES in Figure 3d shows a shift in the kinetic energy distribution over a very short time scale (highlighted by the red dashed arrow), in contrast to ES. The distribution starts at a confined area centered around 10,000 cm^–1^ before dispersing to a lower kinetic energy diffuse distribution within 100–200 fs and decaying from there. This photoelectron kinetic energy shift, together with the ultrafast ∼0.3 ps excited-state lifetime, further implies dramatic changes in energy along the unstable S_1_(ππ*) PES and onto the ground electronic state as the molecule relaxes. Additionally, the eKE spectra show no visible long-lived component, implying that the likelihood of a trapped excited-state population in DES is negligible when excited to the S_1_(ππ*) state in the gas phase.

We close our discussion of DES by determining its IP, as this has not been reported elsewhere in the literature. An eKE_max_ of approximately 12,750 cm^–1^ is observed from the eKE false color heatmap in Figure 3d. Using eq 1, an estimate for the IP of DES is found to be 59,686 cm^–1^. This is slightly lower but very similar to that seen for ES and MS.^22^ It agrees well with the computational IP values (60,331 cm^–1^ at the DFT level and 57,427 cm^–1^ at the XMS-CASPT2 level). The similarity of the IP between ES and DES is not surprising, given their similar HOMO energies (∼0.26 au at DFT level), in line with Koopmans’ theorem.^36^ Therefore, we conclude that symmetrizing the ester moieties across the acrylic double bond in sinapates has little effect on the IP of the molecule.

Bringing the results for ES and DES together, a combination of factors may explain the remarkable difference in their lifetimes. First, the ES S_1_ potential energy surface is much flatter, exhibiting a local minimum and a sloped CI along the isomerization coordinate, which leads to slow excited-state dynamics. In contrast, the DES surface is steep, with no local minimum and a peaked CI along the isomerization coordinate, all factors speeding up the deactivation to the ground state. Lastly, due to the energetic proximity between the S_1_ and S_2_ states in the case of ES, their electronic mixing may also slow down the dynamics.^37^

To conclude, it is evident that symmetrizing the ester moieties across the ES acrylic double bond dramatically impacts the dynamics in the gas phase, as seen for the solution phase, reinforcing the conclusion that this is a fundamental structure–dynamics relationship.^18^ In ES, the dynamics extends beyond 1 ns, with evidence of trapped population in the S_1_(ππ*) state, whereas in DES, the relaxation lifetime is more than three orders of magnitude faster, with the molecule fully relaxing by ∼1 ps. The present gas-phase data, along with prior studies in solution, strongly suggest that upon excitation to the S_1_(ππ*) state, both molecules undergo IVR along the S_1_(ππ*) PES and toward a CI with the ground electronic state, S_0_. The path along the S_1_(ππ*) state PES is likely to involve a torsion around the acrylic double bond proposed previously in the solution phase for these molecules. With the TR-PE spectroscopy data, we observed unprecedented detail into the changes in potential energy as these molecules relax, indicating small changes for ES but dramatic changes for DES. Furthermore, the incorporation of TR-PE spectroscopy ultimately provided a rationale for the drastic lifetime changes between the two molecules and has gone some way to dismiss the involvement of a triplet state in the ultrafast relaxation of ES in the gas phase. That said, this does not rule out the possibility that a portion of the trapped population may reach a triplet state after 1 ns.

Overall, this fundamental understanding of the impact of symmetrizing sinapate esters around the acrylic double bond provides crucial insights for developing symmetrical biomimetic photothermal molecules. It is a perfect example of how an intuitively simple functionalization of a nature-inspired photothermal molecule can dramatically improve its efficacy to dissipate energy through nonradiative decay pathways. This conclusion contributes to a deeper understanding of how different functionalizations of biomimetic photothermal molecules affect the dissipation of their absorbed energy from a fundamental level, and it is helpful for the development of future technologies that rely on molecules able to convert light into heat. A possible future approach for development could involve further substitution around the acrylic double bond to study effects, such as steric interactions.

## Experimental Methods

The experimental setup has been described in detail elsewhere.^28,38,39^ Briefly, a Ti:sapphire oscillator (Spectra-Physics Tsunami) and regenerative amplifier (Spectra-Physics Spitfire XP) produce ∼40 fs laser pulses at a rate of 1 kHz and centered around 800 nm. The output laser beam is ∼3 W and is subsequently split into three equal ∼1 W parts. Two of these beams are used for this experiment. The first 1 W part was used to pump an optical parametric amplifier (Light Conversion TOPAS-C) producing the pump pulse, centered at either 322 or 325 nm to photoexcite ES or DES, respectively, to their S_1_(ππ*) state. The second 1 W part either was used to pump a second TOPAS-C to generate the 240 nm probe or was successively frequency converted using a series of type I, type II, and type I β-barium borate crystals to produce the 200 nm probe. A temporal delay between the pump and probe was generated though varying the path length of the pump beam with respect to the probe beam using a hollow UV-enhanced aluminum retroreflector mounted on a motorized delay stage. The polarization of the pump and probe were parallel to each other and in the plane of the detector. Upon changing the probe to magic angle (54.7°) with respect to the pump, the dynamics were unaffected, indicating the absence of rotational artifacts (Figure S7). Laser powers were set to ensure single photon dynamics.

The pump and probe intersected a molecular beam produced by seeding either ES heated to 150 °C or DES heated to 170 °C into 1.5 bar helium (see references for synthesis).^17,18^ This gaseous mixture was expanded into vacuum via an Even-Lavie solenoid valve and was subsequently passed through a 2 mm diameter skimmer.^40,41^

For TR-IY, the photoions produced at the point of pump–probe laser intersection are accelerated, via an electric field, toward a detector consisting of a microchannel plate (MCP) coupled with a metal anode detector (Del Mar Photonics MCP-MA25/2). The detector’s output was measured by a digital oscilloscope (LeCroy LT372 Waverunner), and ion-signal relating to either ES^+^ or DES^+^ was recorded as a function of pump–probe time delay (Δ*t*), thus creating the TR-IY transients.

A velocity map imaging setup, based on a design by Eppink and Parker, was used to monitor the corresponding photoelectrons.^42^ The photoelectrons were accelerated and focused onto a position-sensitive detector consisting of two MCPs coupled to a phosphor screen (Photek VID-240) and imaged by a CCD camera (Basler A-312f). With this configuration, electrons with the same initial velocity are mapped onto the same radial position on the detector. The resulting two-dimensional images were used to reconstruct the original three-dimensional Newton sphere via a polar onion peeling algorithm, from which the desired one-dimensional photoelectron spectrum was derived.^43^ The detector was calibrated from scaling xenon’s photoelectron spectrum to coincide with the energy of its well-known ionization states.^44^ Integrating the one-dimensional photoelectron spectra as a function of Δ*t* produced TR-PE transients.

Dynamical information was extracted using an exponential decay model convoluted with a Gaussian instrument response; more details regarding the fitting model can be found in the Supporting Information. The instrument response was estimated from the cross-correlation of the pump and probe with ammonia (see Figure S8). The instrument response was determined to be ∼110 and ∼175 fs for the 240 and 200 nm probe setups, respectively.

## Computational Methods

The ground state of ethyl sinapate (ES) and diethyl sinapate (DES) was optimized at the CAM-B3LYP/cc-pVDZ level in the gas phase.^45^ The most stable conformer was selected for further multiconfigurational calculations for both molecules. For optimizations of S_1_ and S_2_ states and conical intersections, state-average complete active space self-consistent field (SA-CASSCF)^46^ was used. The energies were subsequently computed using extended multistate complete active space second-order perturbation (XMS-CASPT2).^47^ All calculations used 6-31G(d) basis set, no IPEA shift,^48^ and an imaginary shift^49^ of 0.1 au. All DFT calculations were performed using Gaussian 16 Revision A.03.^50^ All CASSCF/XMS-CASPT2 calculations were done using OpenMolcas v.19.11 (tag 283-ge7efbbb).^51^

For ES, an active space composed by 6 electrons in 6 orbitals (see Figure S11) and 4 states in the average was selected, referred to as SA4-CASSCF(6,6). For DES, we used an active space comprising 8 electrons in 7 orbitals and 3 states in the average (see Figure S12). For conical intersections only two states were used. Linear interpolated geometries in the internal coordinates (LIIC) were performed at the same level of theory for ES. Ionization potentials for ES and DES were computed at S_0_ geometries (optimized with CAM-B3LYP/cc-pVDZ) using (i) CASSCF/XMS-CASPT2, as described above, and (ii) DFT level, using CAM-B3LYP/aug-cc-pVDZ.

## Supporting Information Available

Additional figures and fitting model information; (Figure S1) TR-PE eKE spectra; (Figures S2 and S3) time-resolved β_2_ anisotropy parameter heatmaps; (Figure S4) pump wavelength dependence study on DES; (Figure S5) TR-PE eKE spectra; (Figure S6) parallel and sequential fitting attempts; (Figure S7) magic angle TR-IY; (Figure S8) cross-correlation measurements; computational calculations; (Figures S9–S16 and Tables S1 and S2) molecular geometries, orbitals and energies, active spaces, conical intersection characterization, linear interpolated coordinates in internal coordinates (PDF)
Transparent Peer Review report available (PDF)
