# Supplementary material for: Understanding the Impact of Symmetrical Substitution on the Photodynamics of Sinapate Esters Using Gas-Phase Ultrafast Spectroscopy
Source: J Phys Chem Lett. 2023 Sep 22;14(39):8771–9. doi: 10.1021/acs.jpclett.3c02134 (PMC10561265; doi:10.1021/acs.jpclett.3c02134)
Supplement: Supplementary file 1 — jz3c02134_si_001.pdf [file jz3c02134_si_001.pdf]

Supporting Information for:

Understanding the Impact of Symmetrical Substitution on the Photodynamics of Sinapate Esters Using Gas-Phase Ultrafast Spectroscopy

Jack Dalton,[1] *Josene M. Toldo,*[2,*] *Florent Allais,*[3,] *Mario Barbatti,*[2,4] *and Vasilios G. Stavros*[1,5,*]

[1] *Department of Chemistry, University of Warwick, Gibbet Hill Road, Coventry, CV4 7AL, UK.*

[2] *Aix Marseille Université, CNRS, ICR, Marseille, France.*

[3] *URD Agro-Biotechnologies Industrielles, CEBB, AgroParisTech, 51110 Pomacle, France.*

[4] *Institut Universitaire de France, 75231 Paris, France.*

[5] *School of Chemistry, University of Birmingham, Birmingham, B15 2TT, UK.*

*** Correspondence: josene-maria.toldo@univ-amu.fr (J.M.T.); v.stavros@bham.ac.uk (V.G.S.).*

1. Experimental Results

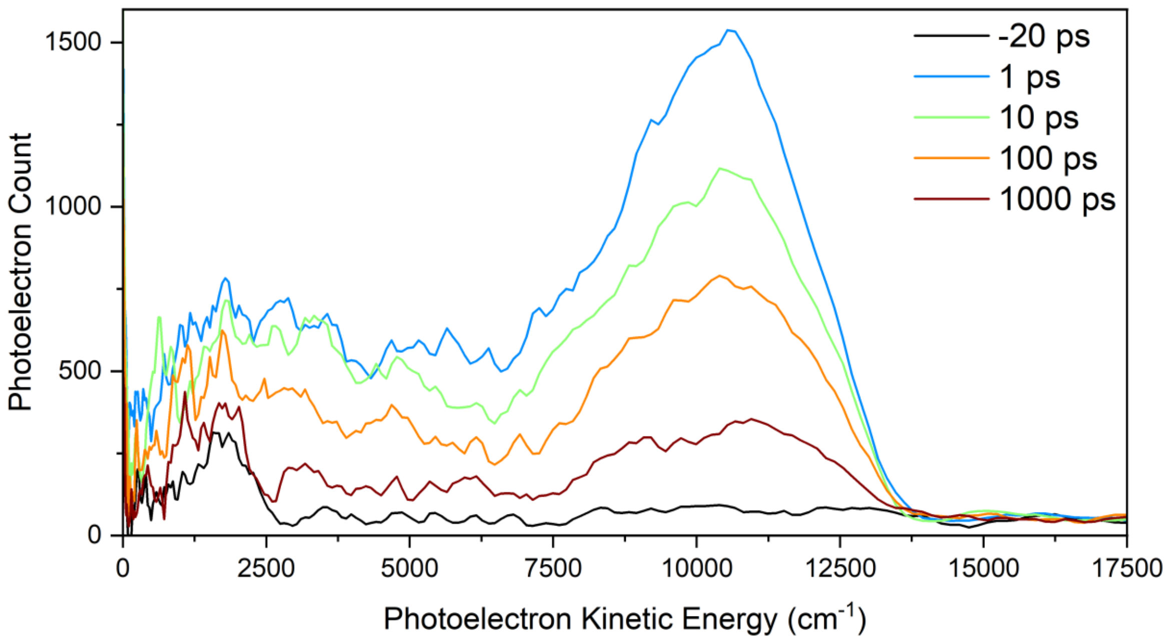

Figure S1. Time-resolved photoelectron (TR-PE) electron kinetic energy (eKE) spectra at select time delays for ethyl sinapate (ES) pumped at 322 nm and probed at 240 nm. The pump and probe are parallel with respect to one another and in the plane of the detector. The eKE was smoothed with a moving average of 4.

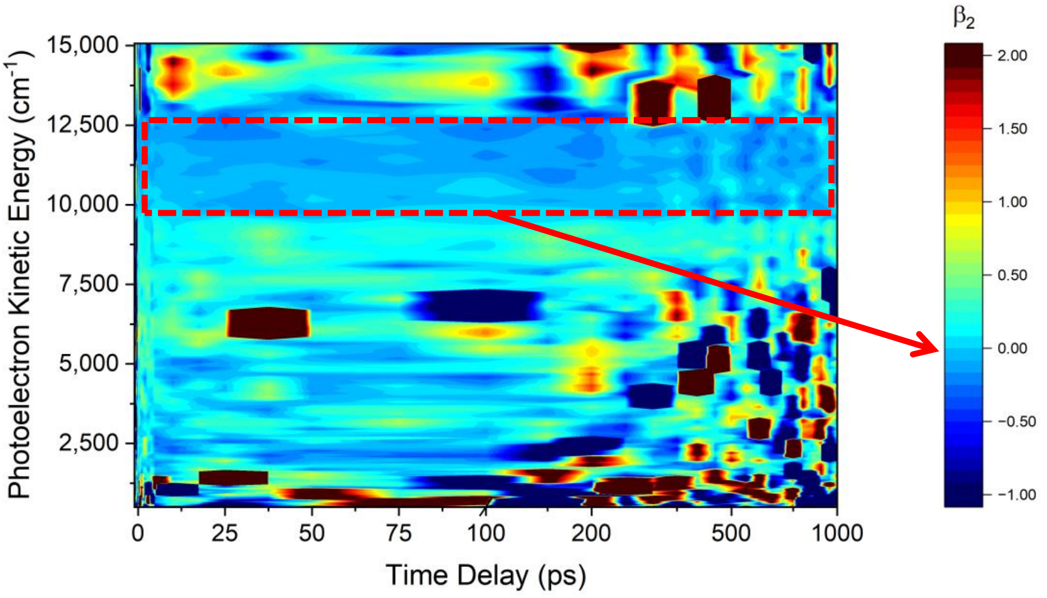

Figure S2. TR-PE eKE false colour heatmap showing the β_2 anisotropy parameter for photoelectron angular distribution of ES pumped at 322 nm and probed at 240 nm. The pump and probe are parallel with respect to one another and in the plane of the detector. The eKE was smoothed with a moving average of 4.

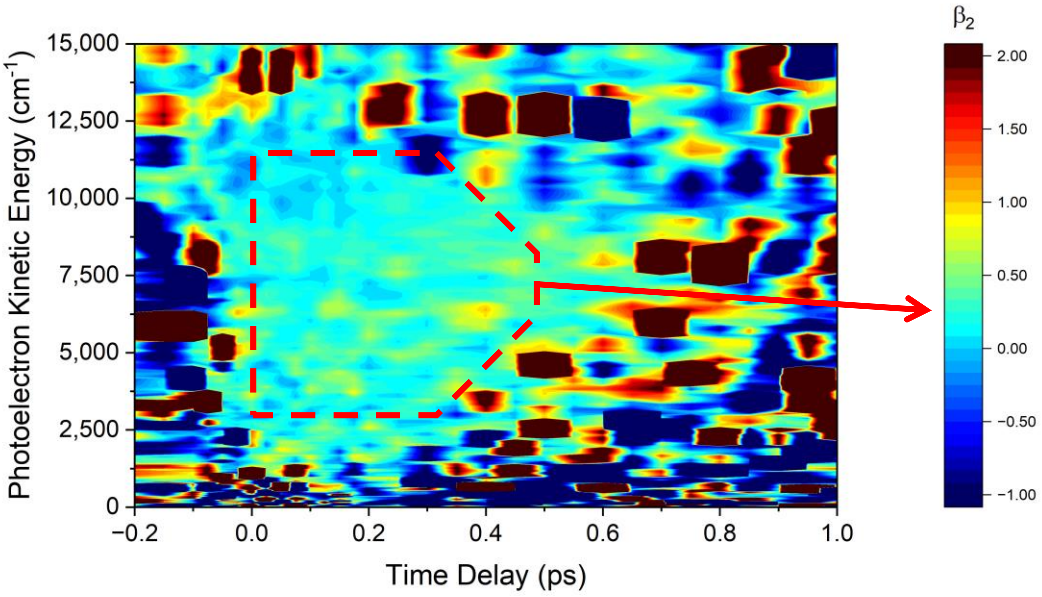

Figure S3. TR-PE eKE false colour heatmap showing the β_2 anisotropy parameter for photoelectron angular distribution of diethyl sinapate (DES) pumped at 325 nm and probed at 240 nm. The pump and probe are parallel with respect to one another and in the plane of the detector. The eKE was smoothed with a moving average of 4.

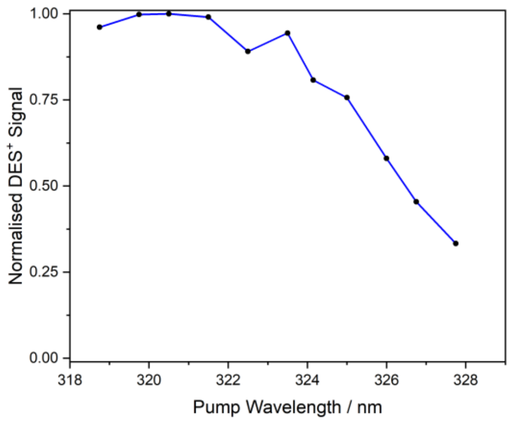

Figure S4. Pump wavelength dependence study on DES. The pump wavelength was scanned, and the DES parent ion signal was recorded. The pump power remained constant across all pump wavelengths.

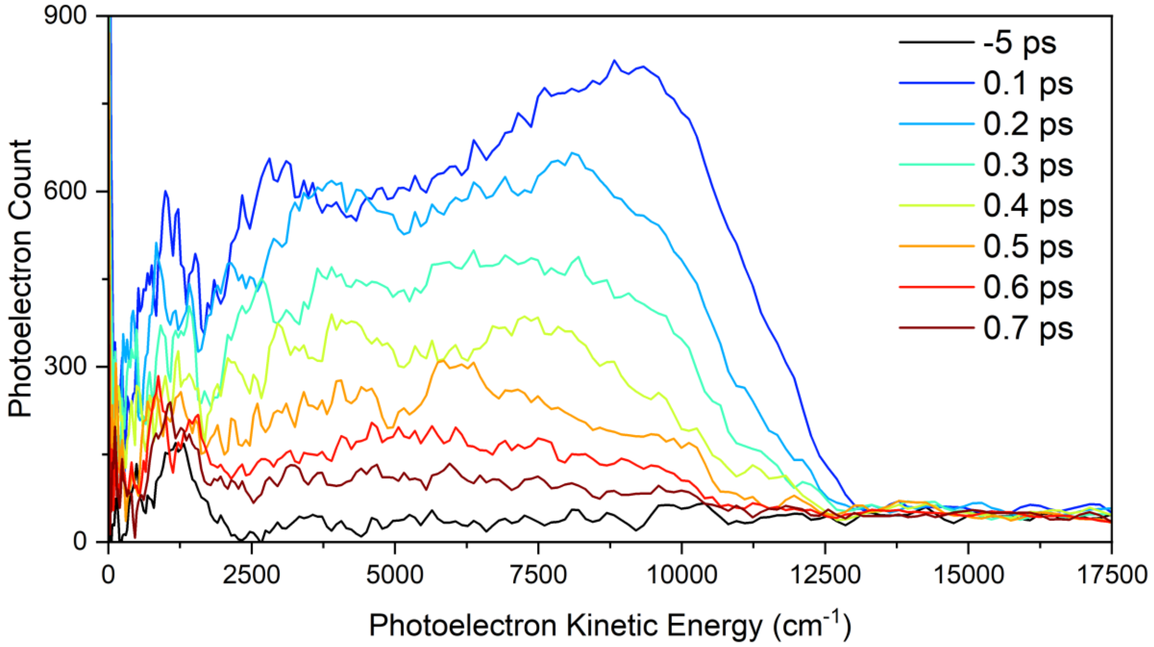

Figure S5. TR-PE eKE spectra at select time delays for DES pumped at 325 nm and probed at 240 nm. The pump and probe are parallel with respect to one another and in the plane of the detector. The eKE was smoothed with a moving average of 4.

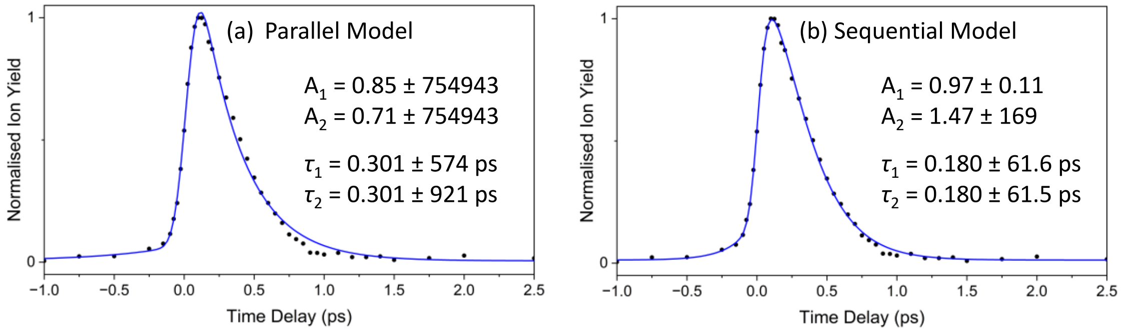

Figure S6. Gas-phase time-resolved ion-yield (TR-IY) transients of DES excited at 325 nm and probed at 240 nm. (a) is fit using a parallel model with 2 exponential decays and (b) is fit with a sequential model with 2 exponential decays. The amplitude of the first (A_1) and second (A_2) decay are displayed along with the lifetimes of the first (τ_1) and second (τ_2) decay – included are the exceptionally large errors produced for each of these values.

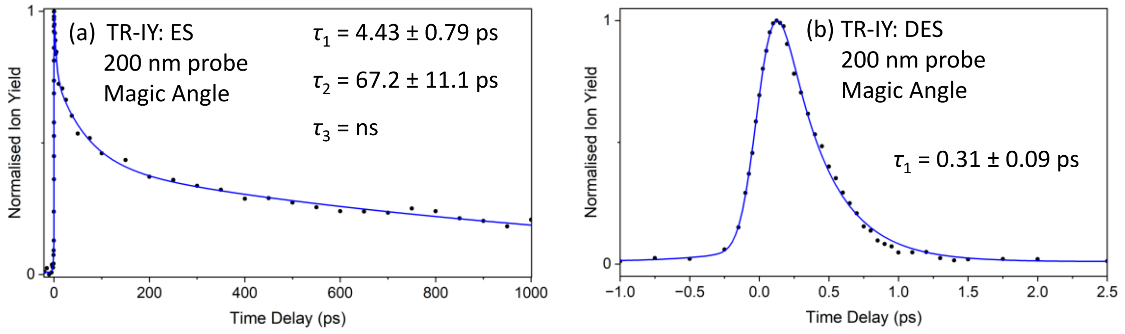

Figure S7. Gas-phase TR-IY transients of ES excited at 322 nm, (a), and DES excited at 325 nm, (b). The probe is 200 nm and held at magic angle (54.7°) with respect to the pump. The blue traces are fits returning time constants shown for the respective plot.

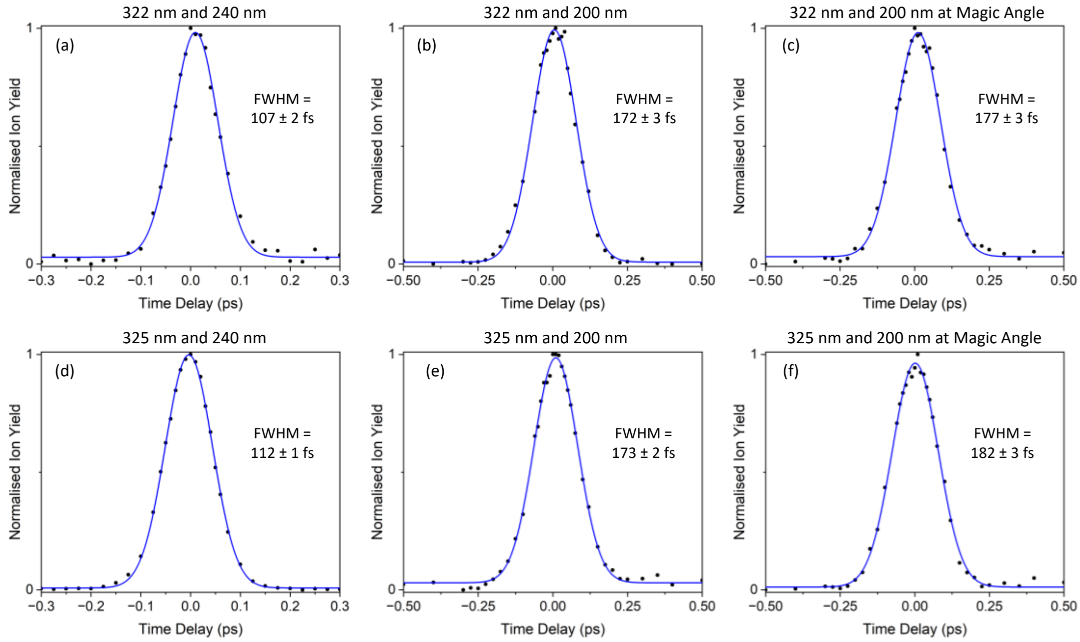

Figure S8. TR-IY cross-correlation with ammonia to estimate the temporal resolution of the experiment. Here, 5% ammonia/helium gas mixture is expanded into vacuum via the Even-Lavie solenoid valve and subsequentially excited and ionised with varying pump-probe time delays. The pump and probe wavelengths are shown above each graph. The polarisation of the pump and probe are parallel with respect to each other and in the plane of the detector for (a), (b), (d) and (e), and at magic angle with respect to each other for (c) and (f). The blue traces are Gaussian fits of the data with the full width half maximum (FWHM) shown for each plot.

Transient Fitting Models

Parallel fitting model. This model consists of a sum of exponential decay functions that are convoluted with a Gaussian function to model the instrument response:

$$S(t) = S_0 + g(t) * \left[\sum_i A_i e^{-k_i t}\right].$$

Here, $t = \Delta t - t_0^{true}, S(t)$ is the time delay dependent signal, S_0 is the signal baseline, $g(t)$ is the Gaussian function, A_i is the amplitude of the i-decay and k_i is the rate of the i-decay – related to the lifetime of the i-decay (τ_i) according to $k_i = 1/\tau_i$.

Sequential fitting model. The following is a model for a system involving 2 sequential processes, 1 and 2:[1]

$$S(t) = S_0 + g(t) * \left[A_1 e^{-k_1 t} + A_2 \frac{k_1}{k_2 - k_1}(e^{-k_1 t} - e^{-k_2 t})\right].$$

2. Computational Results

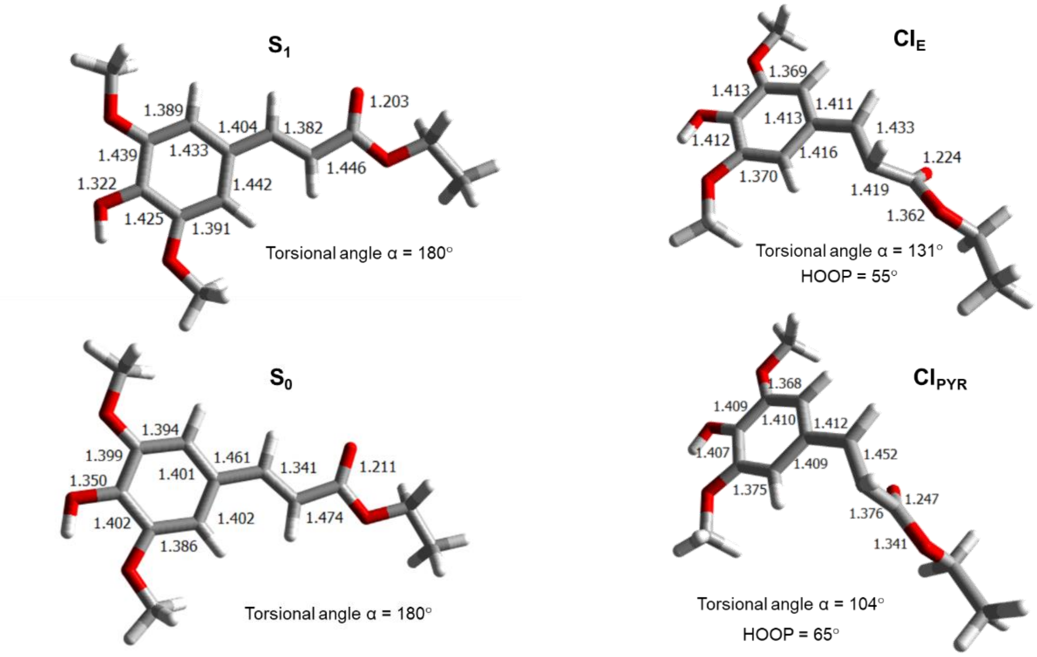

Figure S9. Optimized geometries of ethyl sinapate.

Table S1. Excited state energies and oscillator strengths of ES and DES at XMS-CASPT2//SA-CASSCF level.

				**Ethyl Sinapate**				
	**S_0 geom**		**S_1 geom (V state)**		**S_2 geom (V'state)**		**CI_E**	**CI_{PYR}**
	**Energy (eV)**	***f***	**Energy (eV)**	***f***	**Energy (eV)**	***f***	**Energy (eV)**	**Energy (eV)**
**S_0**	0.00		0.33		0.33		3.65	3.29
**S_1**	4.34	0.415	4.12	0.531	4.36	0.004	3.73	3.41
**S_2**	4.36	0.043	4.30	0.009	4.53	0.419		
**S_3**	6.29	0.046	5.62	0.038	6.30	0.050		
				**Diethyl sinapate**				
	**S_0 geom**						**CI_{DES}**	
**S_0**	0.00						2.50	
**S_1**	3.96	0.434					2.75	
**S_2**	4.16	0.077						

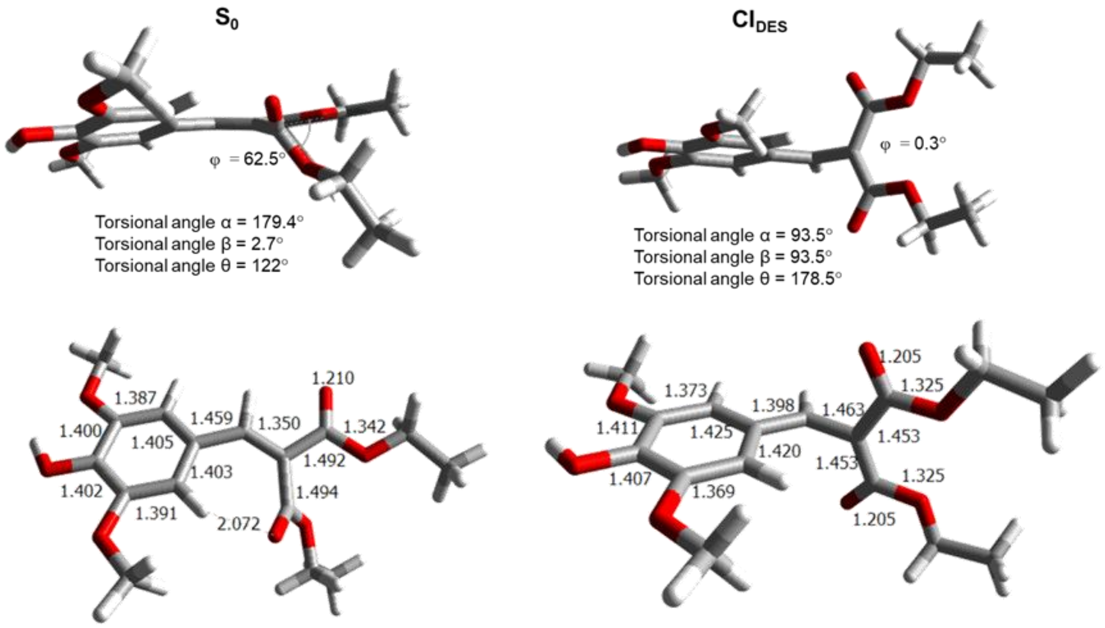

Figure S10. Optimized geometries of diethyl sinapate.

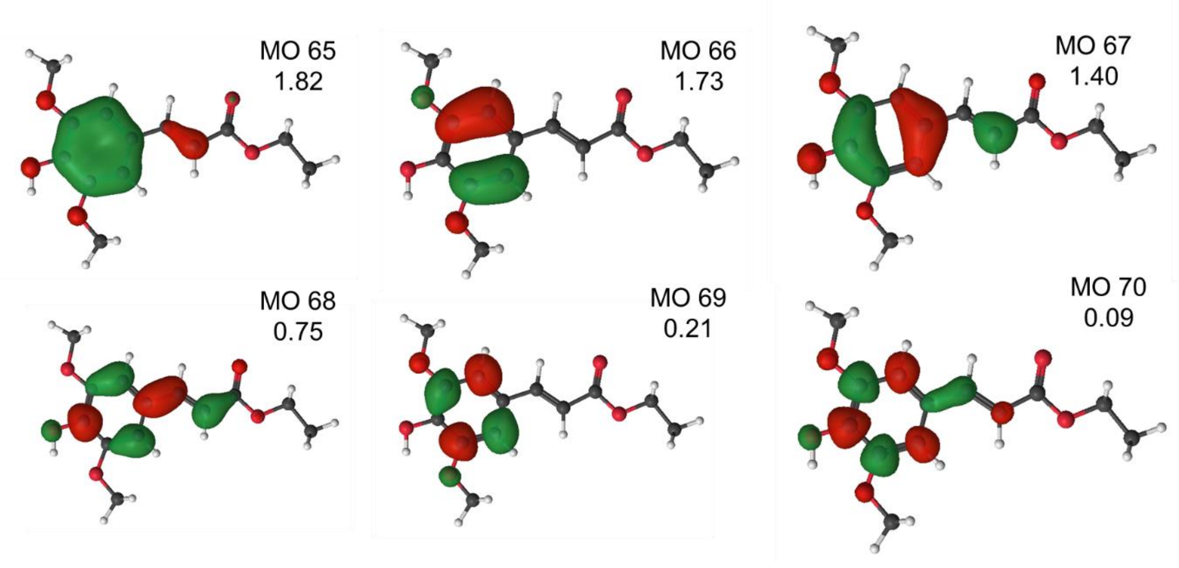

Figure S11. Active space used for ethyl sinapate at SA4-CASSCF(6,6)/6-31G(d) level. The occupation number of the natural orbitals is also given.

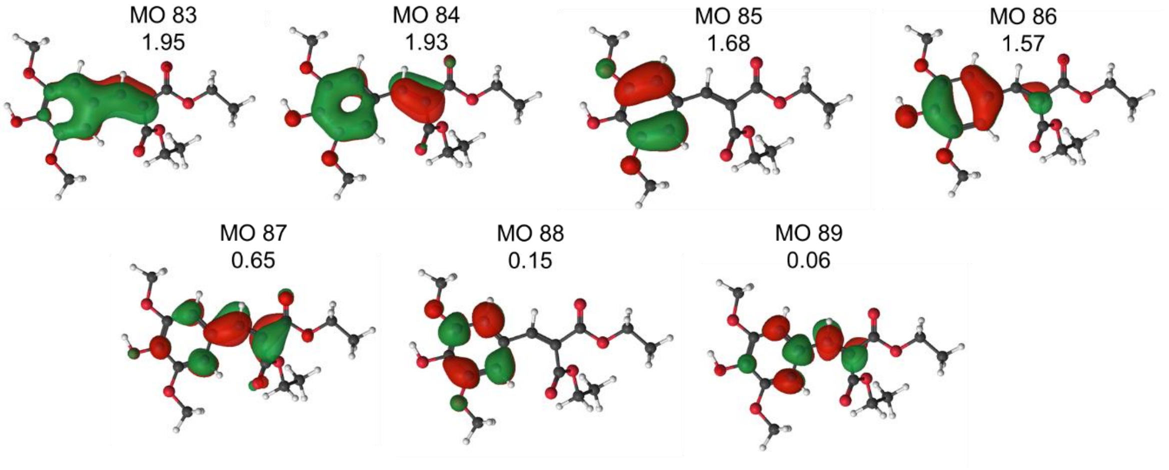

Figure S12. Active space used for diethyl sinapate at SA3-CASSCF(8,7)/6-31G(d) level. The occupation number of the natural orbitals is also given.

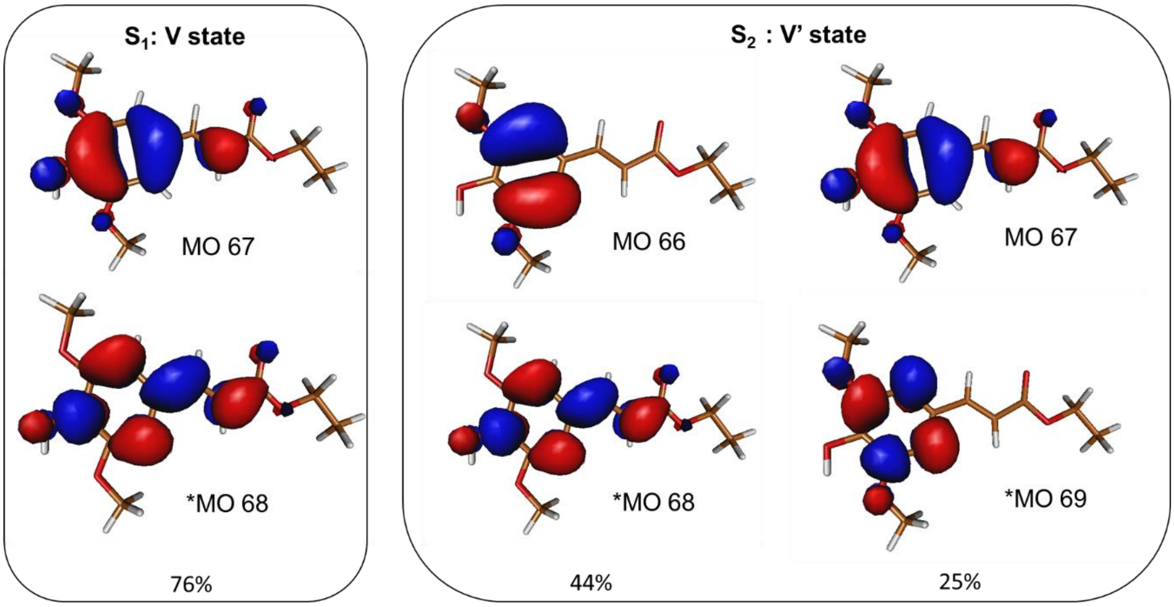

Figure S13. State character and main contribution (in %) of the electronic transition for the two lowest excited states of ethyl sinapate at CASSCF/XMS-CASPT2 level.

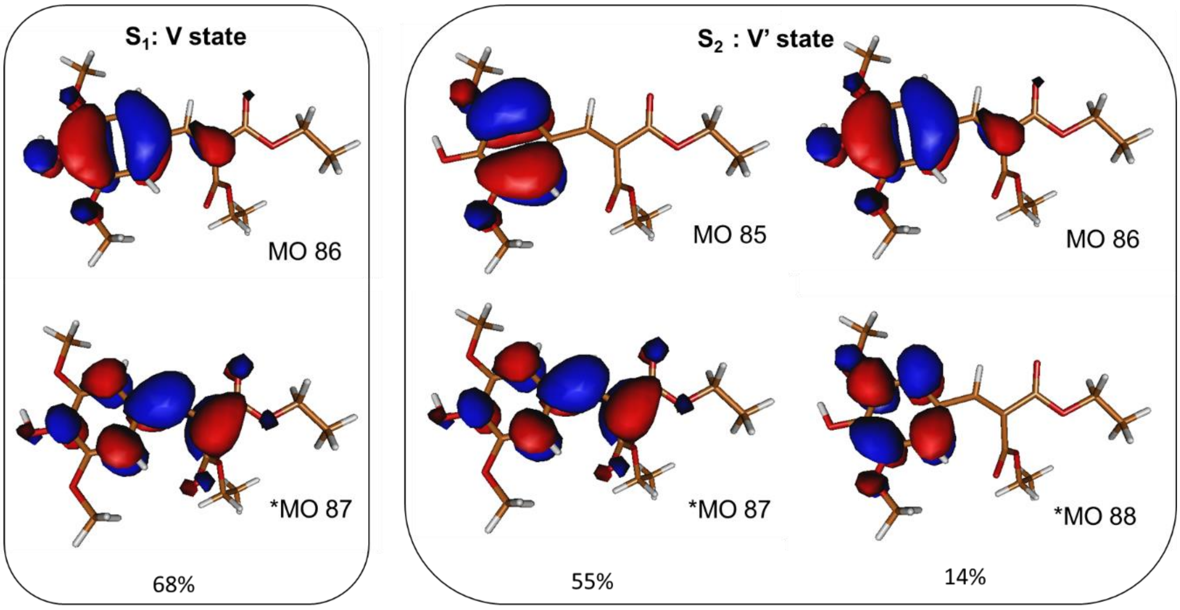

Figure S14. State character and main contribution (in %) of the electronic transition for the two lowest excited states of diethyl sinapate.

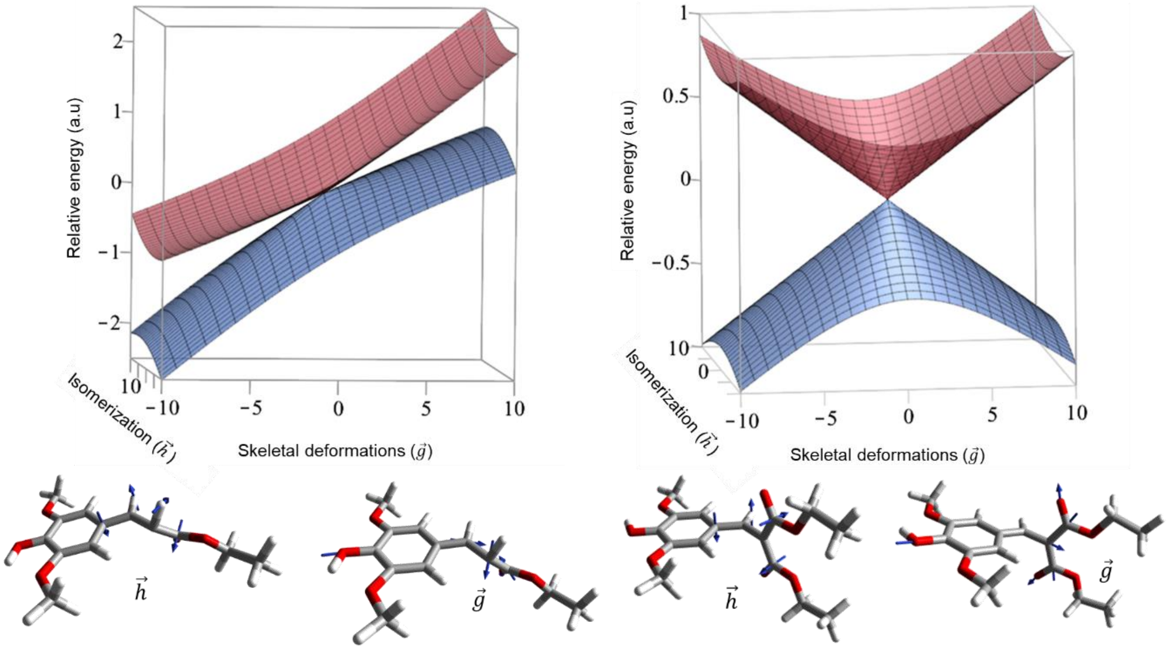

Figure S15. Topography of the conical intersection characterization of ethyl sinapate (left) and diethyl sinapate (right). The cuts along the difference gradient vector ($\vec{g}$) and the nonadiabatic coupling vector ($\vec{h}$) are highlighted on the bottom. The blue arrows in the molecules represent the direction of the displacement modes, which mainly contribute to the gradient difference vector and to the nonadiabatic coupling vector. The gradient difference corresponds to skeletal deformations mainly involving bond-length alternation and HOOP mode for ES. The $\vec{h}$ is dominated by the torsional motion.

Table S2. Topography parameters for the S_1/S_0 conical intersections (in atomic units and degrees). Sloped intersection (P > 1); Peaked (P < 1); Single path (B > 1); bifurcating (B < 1).

Structure	P	B	δgh (H/au)	Δ_{gh}	σ (H/au)	θ_s	$S^{AB}x$ (H/au)	$S^{AB}y$ (H/au)
CI_E	4.98	3.72	0.0599	0.2560	2.4873	0.124	0.1477	0.0184
CI_{PYR}	1.85	1.71	0.0688	0.5192	1.6724	0.043	0.1150	0.0049
CI_{DES}	0.00	0.23	0.0657	0.5115	0.0855	0.017	0.0056	0.0001

* δgh is the strength or pitch of the branching plane, given by the sum of energy difference in x and y directions; the asymmetry of the intersection is given by Δ_{gh}. $\Delta_{gh} = 0$ corresponds to a completely symmetric CI where the degeneracy is raised at the same rate for displacements along $\vec{g}$ and $\vec{h}$. σ is the relative tilt, i.e. how much the intersection plane is tilted with respect to the xy plane. The tilt angle (θ) defines the angles between these two planes.

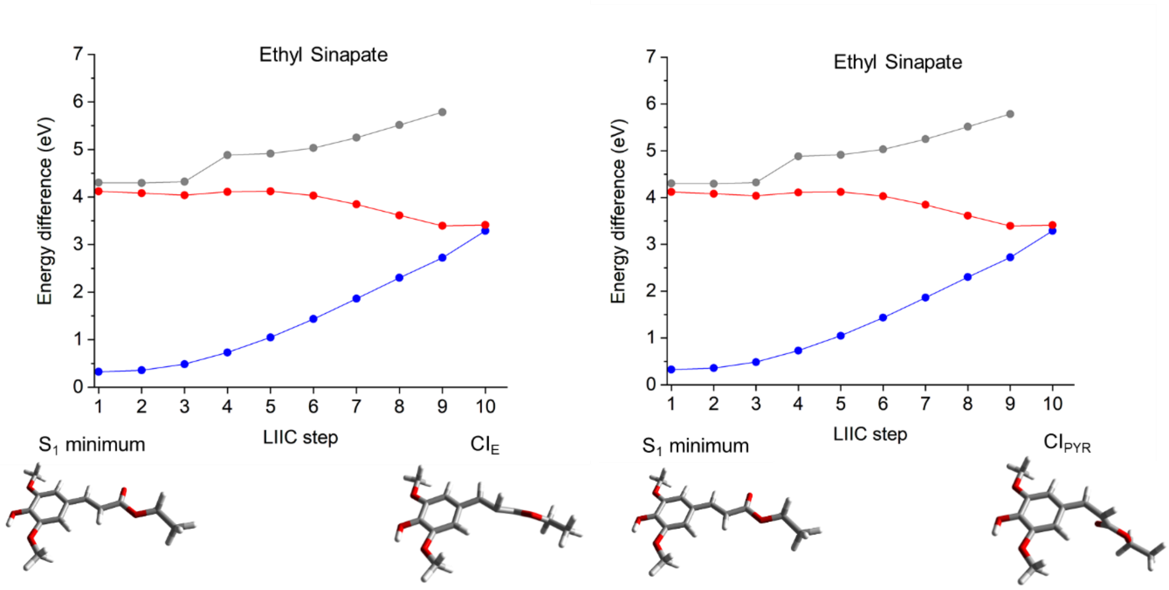

Figure S16. Linear interpolation in internal coordinates from the S_1 minimum to CI_E (left) and to CI_{PYR} (right) at XMS-CASPT2(6,6) level. Blue, red, and grey lines represent the S_0, S_1 and S_2 states respectively.

References

(1) Chatterley, A. S.; Roberts, G. M.; Stavros, V. G. Timescales for Adiabatic Photodissociation Dynamics from the ÃÃ State of Ammonia. *J. Chem. Phys.* **2013**, *139*, 34318.
