# Supplementary material for: Understanding the Impact of Symmetrical Substitution on the Photodynamics of Sinapate Esters Using Gas-Phase Ultrafast Spectroscopy
Source: J Phys Chem Lett. 2023 Sep 22;14(39):8771–9. doi: 10.1021/acs.jpclett.3c02134 (PMC10561265; doi:10.1021/acs.jpclett.3c02134)
Supplement: Supplementary file 2 — jz3c02134_si_002.pdf [file jz3c02134_si_002.pdf]

jz-2023-02134b.R1

Name: Peer Review Information for "Understanding the Impact of Symmetrical Substitution on the Photodynamics of Sinapate Esters Using Gas-Phase Ultrafast Spectroscopy"

First Round of Reviewer Comments

Reviewer: 1

Comments to the Author

This paper reports on the use of time-resolved photoionization-based spectroscopy to investigate the non-adiabatic dynamics operating in ethyl- and diethyl-sinapate in the gas phase. There are also extensive supporting theory calculations. The study shows clear differences in relaxation timescales between the two species investigated and argues that this is of interest for photothermal materials. The data appears to be of good quality and experimental/theoretical procedures and analysis, in general, seem to have been carefully and thoughtfully executed – as would be expected from the high-caliber research groups involved. My overarching issue, however, is that the paper quickly becomes very frustrating to read due to the numerous references to material contained in the Supporting Information (SI). This contains no fewer than 17 figures (plus two tables), several of which help contribute to following the general scientific narrative. In my opinion this is not really the point of SI, which should be reserved for things like large tables of data and/or additional technical information that may be of interest to the more specialist reader. Overall, it feels like this work is being squeezed into a letter format to which it is not well-suited. Furthermore, I also believe that the work does not have quite the required level of novelty and significance for publication in JPC Lett. – especially given some of the previously published dynamics studies on these systems. My recommendation is therefore that the authors consider extending the length of the manuscript to produce a clearer narrative flow that is easier for the reader to digest, and then re-submit to a long-form journal (e.g. JPC A).

Reviewer: 2

Title: Understanding the Impact of Symmetrical Substitution on the Photodynamics of Sinapate Esters Using Gas-Phase Ultrafast Spectroscopy
Authors: J. Dalton, J. Toldo, F. Allais, M. Barbatti, and V. Stavros

This manuscript describes a combined experimental and theoretical investigation of the photodynamics of two closely related sinapate esters in the gas phase. The authors use a combination of time-resolved ion yield (TR-IY) and time=resolved photoelectron (TR-PE) spectra to map out the photodynamics over the 100 fs to 1 ns timescale. The molecules are brought into the gas phase by heating and expanded into vacuum. Using a pump-probe scheme, the molecules are excited in the UV quite close to their

presumed electronic origins (~322-325 nm) and ionized with a delayed fs pulse either at 240 nm or 200 nm. The striking result that forms the main theme of the paper is that diethyl sinapate (DES) decays back to the ground state on a timescale almost 1000-fold faster than ethyl sinapate.

I think this is a very nice piece of work that deserves publication in JPC Letters. However, there are several points where further clarification are needed.

(1) I find the names 'ethyl sinapate' and 'diethyl sinapate' a bit misleading. The difference between the former and latter is not simply that there are two ethyl groups in the latter, but that there are two ethyl ester substituents. What to do about this is unclear to me.
(2) Throughout the manuscript the authors state that it is the symmetrization of the ethyl ester substituents that is key. However, the authors also argue that this group doesn't change the UV absorption wavelength nor the ionization potential. I wonder whether substitution of a second group with equal steric bulk to the second ethyl ester might be all that is needed to accomplish the speed-up in internal conversion, and that it is rather inconsequential that it is an ethyl ester group. The authors should comment on this and modify their arguments about symmetrization if they think it is warranted.
(3) The big experimental results come out of the comparison between Figures 2 and 3. This reviewer didn't notice the change in the time axis between the two figures for quite a while, as this was not emphasized when Figure 3 was introduced. I would make this a far stronger part of the description of Figure 3 so the reader won't miss it.
(4) The arguments made to understand ethyl sinapate's longer lifetime seem rather weak. Can the authors provide a connection with previous data in which the shape of the conical intersection has this kind of dramatic effect on the excited state lifetime? Aren't we to deduce on this basis that the molecule recrosses the conical intersection region in ES about 1000 times before internal conversion to the ground state? Would wave packet dynamics bear out this idea of S_0-S_1 up-funneling? It would have thought that the much higher density of states in S_0 would prevent up-funneling.
(5) Related to #4: Couldn't it also be possible that the excitation bandwidth creates some excited state species that are below the barrier to the conical intersection and are slowed down by spending more time in the Franck-Condon region?
(6) Can the authors map out even a qualitative bandshape for the electronic excitation of ES and DES in the gas phase just to convince us that the excitation enters the S_1 state with similar excess energies in the two molecules?
(7) There are some points in the manuscript where unusual phrasings are used:
    a. Abstract: 'broken down into their core fundamentals'.
    b. P. 2., line 45: 'increasing the number of acquired photons to convert to heat'.
    c. P. 3, line 50: 'how the photodynamics acutely change…'
    d.

Author's Response to Peer Review Comments:

September 4th, 2023

Subject: Submission of the revised manuscript entitled "Understanding the Impact of Symmetrical Substitution on the Photodynamics of Sinapate Esters Using Gas-Phase Ultrafast Spectroscopy"

By Jack Dalton,1 Josene M. Toldo,2,* Florent Allais,3 Mario Barbatti,2,4 and Vasilios G. Stavros1,5,*

1 Department of Chemistry, University of Warwick, Gibbet Hill Road, Coventry, CV4 7AL, UK.

2 Aix Marseille Université, CNRS, ICR, Marseille, France.

3 URD Agro-Biotechnologies Industrielles, CEBB, AgroParisTech, 51110 Pomacle, France.

4 Institut Universitaire de France, 75231 Paris, France.

5 School of Chemistry, University of Birmingham, Birmingham, B15 2TT, UK.

* Correspondence: josene-maria.toldo@univ-amu.fr (J.M.T.); v.stavros@bham.ac.uk (V.G.S).

We would like to submit our revised manuscript entitled "Understanding the Impact of Symmetrical Substitution on the Photodynamics of Sinapate Esters Using Gas-Phase Ultrafast Spectroscopy" for publication in The Journal of Physical Chemistry Letters in the subject category "Physical Insights into Light Interacting with Matter".

We extend our gratitude to the reviewers for their time in assessing our manuscript. The comments have been very helpful, and we are very appreciative of their contributions. We feel their input into our work has improved the quality of our manuscript.

The responses to all the comments have been compiled in a separate document which contains point-by-point colour-coded actions taken in response to the minor concerns raised.

We look forward to hearing from you regarding our revised manuscript. We thank you in advance for your kind consideration and we are happy to respond to any further comments or questions that you may have.

Sincerely,

Vasilios Stavros FRSC

Professor of Physical Chemistry

Journal: The Journal of Physical Chemistry Letters
Manuscript ID: jz-2023-02134b
Original Submission Date: 31-Jul-2023
Title: "Understanding the Impact of Symmetrical Substitution on the Photodynamics of Sinapate Esters Using Gas-Phase Ultrafast Spectroscopy"
Author(s): Dalton, Jack; Toldo, Josene; Allais, Florent; Barbatti, Mario; Stavros, Vasilios

Subject: Response to the reviewers' comments

Reviewer 1

We would like to thank **reviewer 1** for reviewing our paper and expressing their comments. Our responses are documented below with our actions in the revised manuscript highlighted in blue.

This paper reports on the use of time-resolved photoionization-based spectroscopy to investigate the nonadiabatic dynamics operating in ethyl- and diethyl-sinapate in the gas phase. There are also extensive supporting theory calculations. The study shows clear differences in relaxation timescales between the two species investigated and argues that this is of interest for photothermal materials. The data appears to be of good quality and experimental/theoretical procedures and analysis, in general, seem to have been carefully and thoughtfully executed – as would be expected from the high-caliber research groups involved. My overarching issue, however, is that the paper quickly becomes very frustrating to read due to the numerous references to material contained in the Supporting Information (SI). This contains no fewer than 17 figures (plus two tables), several of which help contribute to following the general scientific narrative. In my opinion this is not really the point of SI, which should be reserved for things like large tables of data and/or additional technical information that may be of interest to the more specialist reader. Overall, it feels like this work is being squeezed into a letter format to which it is not well-suited. Furthermore, I also believe that the work does not have quite the required level of novelty and significance for publication in JPC Lett. – especially given some of the previously published dynamics studies on these systems. My recommendation is therefore that the authors consider extending the length of the manuscript to produce a clearer narrative flow that is easier for the reader to digest, and then re-submit to a long-form journal (e.g. JPC A).

Comment R1.1, 'My overarching issue, however, is that the paper quickly becomes very frustrating to read due to the numerous references to material contained in the Supporting Information (SI). This contains no fewer than 17 figures (plus two tables), several of which help contribute to following the general scientific narrative. In my opinion this is not really the point of SI, which should be reserved for things like large tables of data and/or additional technical information that may be of interest to the more specialist reader. Overall, it feels like this work is being squeezed into a letter format to which it is not well-suited.'

Response: We appreciate this reviewer's concern over the large amount of data present in the SI. When originally crafting the manuscript, our main focus was on the experimental work (and still is) supported by theory. As a result, we took the decision to migrate much of the theory results to the supporting information whilst keeping the central theory results in the main manuscript. We have tried to rework some of the text in the main manuscript to stress this and we hope this goes some way to allay the concerns of this reviewer.

Action: To further stress this in the paper the start of the theoretical results now includes the following sentence on page 8:

“The main computational findings are presented in the present manuscript; further supplementary details are presented in figures S9 to S16 and tables S1 and S2.”

Additionally, we have carefully considered mention (and discussion) of all SI figures in the main manuscript and found that by including, in the main manuscript, the torsional angles originally depicted in Fig. S9, this would assist the narrative. As such, these tortional angles have been included in Fig. 1 of the main manuscript (see below) and Fig. S9 has been removed from the SI. To add, all figure references have been updated accordingly.

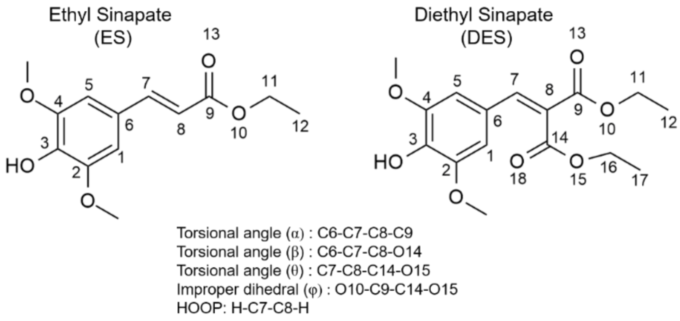

“Figure 1. Chemical structure with atom numbering of ethyl sinapate (ES) and its symmetrical analogue, diethyl sinapate (DES). Below are the main torsional angles discussed *infra*.”

Comment R1.2, ‘Furthermore, I also believe that the work does not have quite the required level of novelty and significance for publication in JPC Lett. – especially given some of the previously published dynamics studies on these systems.’

Response: Whilst we acknowledge this reviewers’ comments, we respectfully disagree. Previous gas-phase work from our group and others has focused largely on time-resolved ion-yield studies which give little insight into the changes in energy a molecule undergoes after photoexcitation. The present work uses time-resolved photoelectron spectroscopy (TR-PES) for the first time to garner insight into the evolution of energy in these systems and determine whether triplet states are involved in the ultrafast relaxation; this was previously speculated to be the case. Additionally, TR-PES has provided a viewing-glass into the the drastic differences in potential energy changes from two seemingly similar sinapates, as they relax following photoexcitation. These novel experimental findings are thoroughly supported by high level theory calculations which in themselves find and demonstrate the importance of conical intersection topography on the relaxation of sinapate molecules.

Action: To further highlight this novelty and significance in the paper, the following sentence has been included when discussing triplet state formation in the gas phase (page 4):

“However, despite these extensive studies, none have monitored the molecule’s potential energy on the ultrafast time scale and thus, cannot confirm triplet state formation within 30 ps.”

Reviewer 2

We would like to thank **reviewer 2** for taking the time to read the manuscript and providing comments and identifying points for improvements. Our responses are documented below with our actions in the revised manuscript highlighted in red.

This manuscript describes a combined experimental and theoretical investigation of the photodynamics of two closely related sinapate esters in the gas phase. The authors use a combination of time-resolved ion yield (TR-IY) and time-resolved photoelectron (TR-PE) spectra to map out the photodynamics over the 100 fs to 1 ns timescale. The molecules are brought into the gas phase by heating and expanded into vacuum. Using a pump-probe scheme, the molecules are excited in the UV quite close to their presumed electronic origins (~322-325 nm) and ionized with a delayed fs pulse either at 240 nm or 200 nm. The striking result that forms the main theme of the paper is that diethyl sinapate (DES) decays back to the ground state on a timescale almost 1000-fold faster than ethyl sinapate.

I think this is a very nice piece of work that deserves publication in JPC Letters. However, there are several points where further clarification are needed.

(1) I find the names 'ethyl sinapate' and 'diethyl sinapate' a bit misleading. The difference between the former and latter is not simply that there are two ethyl groups in the latter, but that there are two ethyl ester substituents. What to do about this is unclear to me.

(2) Throughout the manuscript the authors state that it is the symmetrization of the ethyl ester substituents that is key. However, the authors also argue that this group doesn't change the UV absorption wavelength nor the ionization potential. I wonder whether substitution of a second group with equal steric bulk to the second ethyl ester might be all that is needed to accomplish the speed-up in internal conversion, and that it is rather inconsequential that it is an ethyl ester group. The authors should comment on this and modify their arguments about symmetrization if they think it is warranted.

(3) The big experimental results come out of the comparison between Figures 2 and 3. This reviewer didn't notice the change in the time axis between the two figures for quite a while, as this was not emphasized when Figure 3 was introduced. I would make this a far stronger part of the description of Figure 3 so the reader won't miss it.

(4) The arguments made to understand ethyl sinapate's longer lifetime seem rather weak. Can the authors provide a connection with previous data in which the shape of the conical intersection has this kind of dramatic effect on the excited state lifetime? Aren't we to deduce on this basis that the molecule recrosses the conical intersection region in ES about 1000 times before internal conversion to the ground state? Would wave packet dynamics bear out this idea of S0- S1 up-funneling? It would have thought that the much higher density of states in S0 would prevent up-funneling.

(5) Related to #4: Couldn't it also be possible that the excitation bandwidth creates some excited state species that are below the barrier to the conical intersection and are slowed down by spending more time in the Franck-Condon region?

(6) Can the authors map out even a qualitative bandshape for the electronic excitation of ES and DES in the gas phase just to convince us that the excitation enters the S1 state with similar excess energies in the two molecules?

(7) There are some points in the manuscript where unusual phrasings are used:

a. Abstract: 'broken down into their core fundamentals'.

b. P. 2., line 45: 'increasing the number of acquired photons to convert to heat'.

c. P. 3, line 50: 'how the photodynamics acutely change…'

Comment R2.1: *"I find the names 'ethyl sinapate' and 'diethyl sinapate' a bit misleading. The difference between the former and latter is not simply that there are two ethyl groups in the latter, but that there are two ethyl ester substituents. What to do about this is unclear to me."*

Response: We thank the reviewer for this comment and agree it can be misleading. The full name has now been used in the first mention of the molecule and abbreviated to DES thereafter.

Action: The abstract now includes the following on page 1: "diethyl 2-(4-hydroxy-3,5-dimethoxybenzylidene)malonate (or diethyl sinapate, DES)". The main text now includes the following on page 3: "diethyl 2-(4-hydroxy-3,5-dimethoxybenzylidene)malonate (diethyl sinapate), abbreviated DES hereon".

Comment R2.2: *"Throughout the manuscript the authors state that it is the symmetrization of the ethyl ester substituents that is key. However, the authors also argue that this group doesn't change the UV absorption wavelength nor the ionization potential. I wonder whether substitution of a second group with equal steric bulk to the second ethyl ester might be all that is needed to accomplish the speed-up in internal conversion, and that it is rather inconsequential that it is an ethyl ester group. The authors should comment on this and modify their arguments about symmetrization if they think it is warranted."*

Response: We thank the reviewer for this argument and agree that there may be merit to this that would require further investigation.

Action: In the concluding remarks and future work of the manuscript, we have included the following (Page 15):

> "A possible future approach for development could involve further substitution around the acrylic double bond to study effects such as steric interactions."

Comment R2.3: *"The big experimental results come out of the comparison between Figures 2 and 3. This reviewer didn't notice the change in the time axis between the two figures for quite a while, as this was not emphasized when Figure 3 was introduced. I would make this a far stronger part of the description of Figure 3 so the reader won't miss it."*

Response: We thank the reviewer for highlighting the need to emphasize the drastic change in the time delay scale between Fig. 2 and 3. This has been rectified accordingly.

Action: The caption of Fig. 3 now reads:

> "TR-IY and TR-PE transients of DES photoexcited at 325 nm (note the difference in time delay scale to that in Fig. 2)."

Comment R2.4a: *"The arguments made to understand ethyl sinapate's longer lifetime seem rather weak. Can the authors provide a connection with previous data in which the shape of the conical intersection has this kind of dramatic effect on the excited state lifetime?"*

Response: We do not believe the topography of the conical intersection as an isolated effect determines the lifetime, but a combination of effects.

Action: To make it clearer, we added further clarifying text on page 12:

"The calculations confirm a steep downhill pathway from the initially excited S_1 state towards the CI with the ground state (Fig. 4), which, combined with a favourable peaked CI (SI Fig. S15), can explain the dramatically shorter lifetime of DES."

Response continued: Nevertheless, the effect of the local topography on the outcome of nonadiabatic transitions can be dramatic, as pointed out by Yarkony (DOI: 10.1063/1.1329644). One classic example of this dramatic effect is the retinal protonated Schiff bases, by Todd Martínez and co-workers, where they explain the bond selectivity (energetic considerations cannot explain that) to the local topography of the conical intersection (DOI: 10.1073/pnas.032658099). Furthermore, other examples are discussed in a more recent paper (DOI: 10.1039/D0CP03464A) where Farfan *et al.* show that the topography of the conical intersection determines molecular properties such as photostability, photoreactivity, excited-state lifetime, reaction rate, and product yield. We mention these references (30-32) at the bottom of page 9.

Action: We have also removed the part of the sentence "implying larger internal conversion times" on page 10, which now reads:

"On the other hand, in a sloped CI, the population is not strongly directed away from the intersection and, as a result, the probability of $S_0 \rightarrow S_1$ up-funnelling (*i.e.* recross to the excited state) is much larger."

Comment R2.4b: *"Aren't we to deduce on this basis that the molecule recrosses the conical intersection region in ES about 1000 times before internal conversion to the ground state?"*

Response: This is an interesting point; however, it is not a matter of counting how many times the molecule recrosses the conical intersection and how this counting can be attributed to the increase in the lifetime. In the case of a sloped intersection, the wavepacket may vibrate between S_1 and S_0 because it is trapped in a diabatic state (DOI: 10.1063/1.4738960). Therefore, it cannot easily stabilize in the ground state.

Discussing the effect of the conical intersection topography goes beyond our paper's scope. We prefer to limit ourselves to direct the reader to previous works analysing this issue, which we do in page 9 when we mention that the conical intersection topography "plays a crucial role in photochemical selectivity and dynamics.[30–32]" In these recommended references, the reader will find an extensive analysis of the topography effect. In particular, in Martinez and co-workers' paper (DOI: 10.1073/pnas.032658099), they explain that *different topographies "are expected to result in quite different dynamics. On the upper (S_1) PES, the peaked CI is more effective than the sloped CI in directing (i.e., 'funnelling') population to the point of intersection. On the lower (S_0) PES, the peaked CI is more effective than the sloped CI in directing population away from the intersection, thereby reducing the probability of $S_0 \rightarrow S_1$ 'up-funnelling'."* They continue: *"In the case of a peaked CI, the excited state trajectory is clearly directed toward the intersection, and once it quenches to the ground state, it is directed away from the CI. When the intersection is sloped, however, the quenched wavepacket is not strongly directed away from the intersection. In fact, one easily sees that it may recross back to the excited state."*

Action: We hope our response above addresses the comment of the reviewer. No further action has been taken to the manuscript.

Comment R2.4c: *"Would wave packet dynamics bear out this idea of S0- S1 up-funneling? It would have thought that the much higher density of states in S0 would prevent up-funneling."*

Response: Wave packet dynamics would confirm the S_0-S_1 up-funnelling because it is usually propagated in the diabatic representation. In this case, the wavepacket would be trapped in a diabatic potential energy surface, implying oscillations between S_1 and S_0. Once more, this discussion is too specific to go into the manuscript.

Action: We hope our response above addresses the comment of the reviewer. No further action has been taken to the manuscript.

Comment R2.5: *"Related to #4: Couldn't it also be possible that the excitation bandwidth creates some excited state species that are below the barrier to the conical intersection and are slowed down by spending more time in the Franck-Condon region?"*

Response: This is a good point raised by this reviewer and something we considered. However, the absence of a fast component in the dynamics akin to DES (*i.e.* 0.3 ps) coupled to the sloped CI revealed by our calculations strongly point towards the conclusions we originally proposed, that is: (1) a portion of excited state population funneling back to the GS through a sloped CI; and (2) residual population trapped in the excited state that persists for greater than the time duration of our experiment.

Comment R2.6: *"Can the authors map out even a qualitative bandshape for the electronic excitation of ES and DES in the gas phase just to convince us that the excitation enters the S1 state with similar excess energies in the two molecules?"*

Response: Experimentally, the excitation wavelength was chosen to be as close to the band origin as possible for both molecules. The band origin for ES was based on REMPI for methyl sinapate and isopropyl sinapate, which have the same 322 nm band origin; we based the excitation of DES by comparing the cyclohexane UV-Vis of ES and DES and saw that the peak absorption for ES is very similar to the band origin from RMEPI and assumed that the peak absorption of DES in cyclohexane would give a good approximation for the band origin in the gas phase. Following this assessment, we carried out a rough wavelength dependence study (SI Fig S4) to confirm we are photoexciting the S_1 state of DES in the gas-phase. Ideally, we would like to have a REMPI for DES (although this is likely difficult due to DES's short lifetime) to be more precise. However, we believe from these assessments that we are on or extremely close to the band origin for both molecules.

We have additional computational evidence that the energy excess alone cannot explain the orders of magnitude difference in the lifetimes of ES and DES. We ran exploratory surface hopping dynamics with TDDFT for the two molecules. We selected the initial conditions at the centre of each molecule's absorption spectrum and, therefore, we were sure that both had the same energy excess. The results of these simulations did not show significantly different lifetimes between the molecules. If the lifetime difference were only energetic (caused by S_1 barriers and surface flatness), our TDDFT dynamics would have captured it. What TDDFT could not do (due to intrinsic method limitations) was properly describe the S_1/S_0 conical intersection topography and internal conversion. Therefore, these exploratory simulations revealed that the energy excess alone was insufficient to explain the experimental results, and we need the effect of the topography. Although the TDDFT dynamics simulations delivered these relevant insights, we consider them too preliminary to be published. For this reason, it is not discussed in the paper.

Action: We hope our response above addresses the comment of the reviewer. No further action has been taken to the manuscript.

Comment R2.7: *"There are some points in the manuscript where unusual phrasings are used:*
a. Abstract: 'broken down into their core fundamentals'.
b. P. 2., line 45: 'increasing the number of acquired photons to convert to heat'.

c. P. 3, line 50: ‘how the photodynamics acutely change…’”

Response: We thank the reviewer for drawing our attention to some unusual phrasing in the manuscript. All three sentences have been rephrased to avoid any possible misunderstanding.

Action: The following changes have been actioned:

a. “… are stripped to their core fundamentals…”
b. “Moreover, to enhance photothermal performance, these systems must have excellent light harvesting capabilities by strongly absorbing light.”
c. “To further develop our foundational understanding of how the photodynamics suddenly change with varying functionalities and, ultimately, environment complexity, we first need to understand the excited state dynamics of ES and DES without the influence of external perturbations — in the gas phase.”
